# Stable Organic Radical for Enhancing Metal–Monolayer–Semiconductor Junction Performance

**DOI:** 10.1021/acsami.2c15690

**Published:** 2023-01-16

**Authors:** J. Alejandro De Sousa, Raphael Pfattner, Diego Gutiérrez, Kilian Jutglar, Stefan T. Bromley, Jaume Veciana, Concepció Rovira, Marta Mas-Torrent, Bruno Fabre, Núria Crivillers

**Affiliations:** † Institut de Ciència de Materials de Barcelona (ICMAB, CSIC), Campus de la UAB s/n, Bellaterra 081093, Spain; ‡ Laboratorio de Electroquímica, Departamento de Química, Facultad de Ciencias, Universidad de los Andes, 5101 Mérida, Venezuela; § Departament de Ciència de Materials i Química Física & Institut de Química Teòrica i Computacional (IQTC), Universitat de Barcelona, c/Martí i Franquès 1-11, 08028 Barcelona, Spain; ∥ Institució Catalana de Recerca i Estudis Avançats (ICREA), E-08010 Barcelona, Spain; ⊥ Univ Rennes, CNRS, ISCR (Institut des Sciences Chimiques de Rennes)-UMR 6226, F-35000 Rennes, France

**Keywords:** organic radicals, charge transport, liquid metal, molecular junctions, silicon functionalization, photodiode

## Abstract

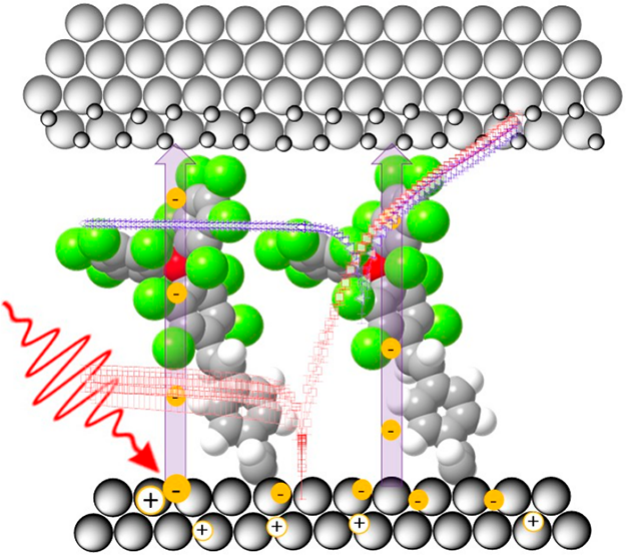

The preparation of monolayers based on an organic radical and its diamagnetic counterpart has been pursued on hydrogen-terminated silicon surfaces. The functional monolayers have been investigated as solid-state metal/monolayer/semiconductor (MmS) junctions showing a characteristic diode behavior which is tuned by the electronic characteristics of the organic molecule. The eutectic gallium–indium liquid metal is used as a top electrode to perform the transport measurements and the results clearly indicate that the SOMO–SUMO molecular orbitals impact the device performance. The junction incorporating the radical shows an almost two orders of magnitude higher rectification ratio (*R*(|*J*_1V_/*J*_–1V_|) = 10^4.04^) in comparison with the nonradical one (*R*(|*J*_1V_/*J*_–1V_|) = 10^2.30^). The high stability of the fabricated MmS allows the system to be interrogated under irradiation, evidencing that at the wavelength where the photon energy is close to the band gap of the radical there is a clear enhancement of the photoresponse. This is translated into an increase of the photosensitivity (*S*_ph_) value from 68.7 to 269.0 mA/W for the nonradical and radical based systems, respectively.

## Introduction

The use of organic molecules in electronics is one of the most promising approaches to develop devices displaying novel and tunable (opto)electronic properties. From one side, organic molecules are already applied in mainstream technologies such as OLED (organic light-emitting diode) displays,^1^ OFETs (organic field-effect transistors),^2,3^ or flexible OPV (organic photovoltaic) cells.^4^ On the other, although molecular-scale electronics is still at an early stage of development for practical applications, the field has grown enormously in the recent years. A significantly improved understanding of the molecule/electrode interfaces, charge transport mechanisms, and the molecular structure/device performance relationship has been reached. Such an in-depth level of understanding has permitted a widening of the complexity of the investigated molecules toward the fabrication of functional molecular junctions.^5^ Commonly, molecular junctions are fabricated sandwiching a single molecule or an assembly of molecules between two metal electrodes. The chemical functionalization of semiconducting substrates, like silicon (Si), has also recently attracted attention due to its relevant technological interest.^6^ The covalent functionalization of Si with organic molecules has opened up the possibility of tuning the electrical properties of the semiconductor beneath^6,7^ as well as of introducing additional functionalities such as redox activity^8^ and biosensing.^9^ Thus, the charge transport through solid-state metal/monolayer/semiconductor (MmS) junctions is of significant interest. However, most of the research done so far on MmS is limited to alkyl molecular monolayers, and very few examples on small conjugated molecules can be found in the literature.^10^ Several parameters such as the molecular dipole, semiconductor dopant type and density, metal electrode characteristics, or even the monolayer quality have a strong impact on the energy level alignment, interface states, and charge rearrangement. In order to understand the electrical properties and thus the (photo)device performance,^11^ it is important to consider and control these different aspects.

Mercury has been widely used as metal contact for the fabrication and electrical characterization of large area molecular junctions because of its mild top contact with the organic materials, resulting in the formation of metal or semiconductor/molecule/Hg junctions.^5^ More recently, the eutectic gallium–indium alloy (EGaIn) has been successfully exploited as a less toxic alternative top contact electrode^12,13^ for studying the charge transport through organic monolayers assembled on metallic surfaces^14^ and oxides^15^ and for the fabrication of other types of devices, like memories.^16,17^ The EGaIn is a low-viscosity liquid at room temperature; it possesses unique properties and has a spontaneously formed thin oxide skin providing a non-Newtonian character. This enables intrinsic flexibility and stretchability combined with high electrical and thermal conductivity, while maintaining the possibility of shaping the EGaIn electrode as a contact tip.^18^ The thickness of the GaO_*x*_, spontaneously formed in air, was reported to be about 0.7 nm^19^ with a typical resistivity of about 4.71 kΩ·cm, which is attributed to the presence of oxygen vacancies in the defective GaO_*x*_ thin layer.^20−22^ This stays in sharp contrast to pure Ga_2_O_3_ (deposited by electron beam evaporation), which is a wide band gap semiconductor (*E*_g_ = 4.8 eV) and exhibits a large electrical resistivity of about 10^12^–10^13^ Ω·cm.^22^ Thus, generally, in a molecular junction, the thin oxide layer contributes with a negligible resistance to the total molecular junction.

Among the different families of molecules investigated in molecular junctions, stable free organic radicals have gained increasing attention over the past few years.^23,24^ Thanks to their open-shell electronic configuration, these molecules are paramagnetic, redox and optically active, which make them appealing species for a variety of applications.^25,26^ Chlorinated trityl radicals, and in particular the perchlorotriphenylmethyl radicals (rad-PTMs), have been shown to be highly stable as active molecular units in molecular junctions.^27−29^ The comparison between junctions incorporating the open-shell (radical) or the closed-shell (nonradical) derivatives allowed to unravel the role of the SOMO–SUMO orbitals (singly occupied molecular orbital-single unoccupied molecular orbital) in the transport mechanisms to be elucidated.^27,30^ From temperature- and chain-length-dependent measurements, it was corroborated that the mechanism of charge transport across the junctions (EGaIn/PTM-monolayer/Au) was direct tunneling and that the SUMO of the radical participated in the transport, effectively lowering the tunneling barrier height.^27^

**Figure 1:**
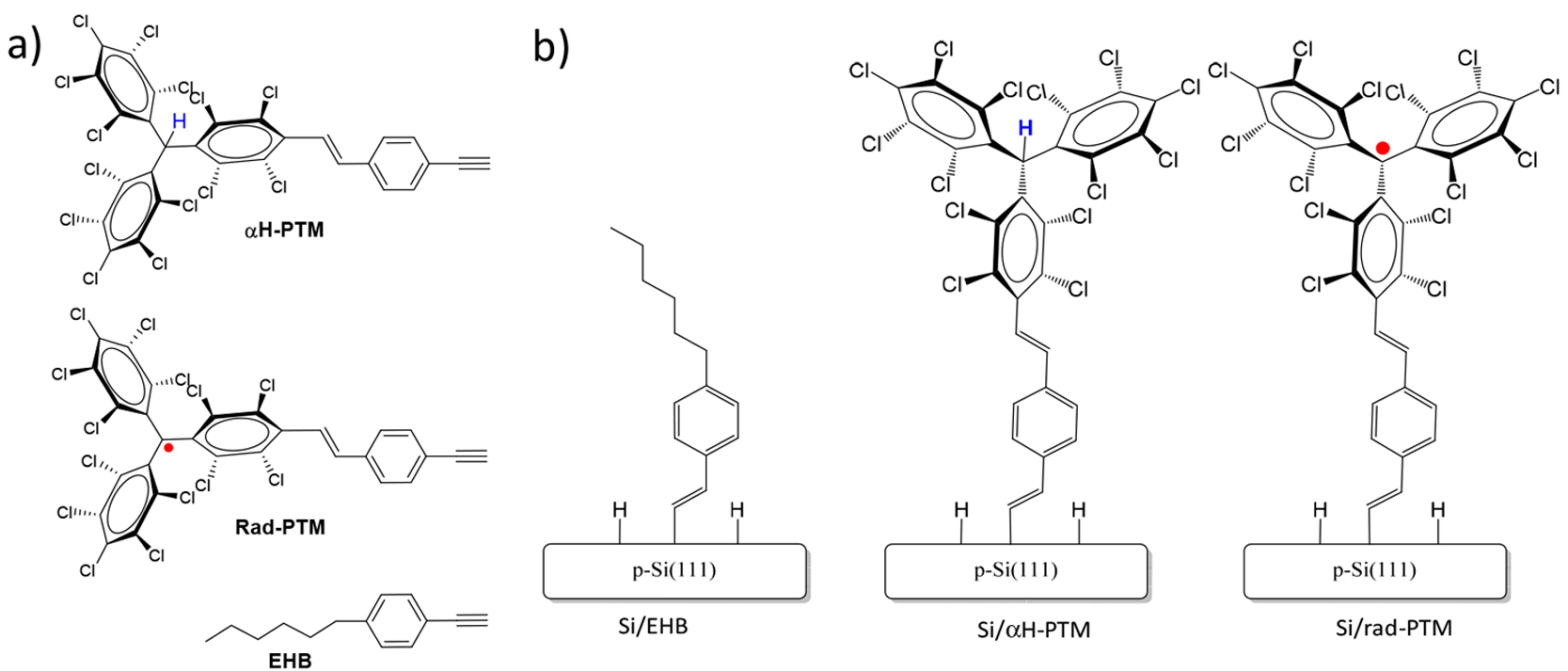
(a) Chemical structure of the PTM derivatives (αH-PTM and rad-PTM) and reference molecule (EHB) employed in this study. (b) Scheme of the three molecular monolayer-modified Si(111) surfaces investigated in this work: Si/EHB (left), Si/αH-PTM (middle), and Si/rad-PTM (right).

Recently, H-terminated p-type silicon (Si–H) was chemically modified through a hydrosilylation route with a PTM radical derivative bearing a terminal alkyne group. Such systems were demonstrated to work as a reversible electrochemical capacitive switch with good stability.^31^ Herein, the charge transport across these systems, employing the open- and closed-shell molecules (rad-PTM and αH-PTM, Figure 1a), is investigated. The monolayers were top-contacted with an EGaIn electrode, leading to a typical MmS junction. Both investigated junctions, i.e., radical and nonradical (αH), showed an expected Schottky-diode behavior for a metal/monolayer/semiconductor molecular junction (i.e., current rectification), but remarkably the radical-based MmS displayed a higher current density in forward bias, which is associated with a more favorable interface energy level alignment due to the presence of the SOMO–SUMO orbitals. At reverse bias, the monolayer contribution into the measured current is negligible. In addition, this interface was evaluated under illumination showing good stability and evidencing a clear effect of the radical on the current response of the junction. To the best of our knowledge, this is the first example of a functional stable organic radical monolayer exploited to modulate the charge transport in MmS Schottky junctions.

## Results and Discussion

### Functionalization and Characterization of Si-H Surfaces

Rad-PTM (open-shell), αH-PTM (closed-shell), and 1-ethynyl-4-hexylbenzene (closed-shell reference sample (EHB)) (Figure 1a) were grafted onto SiO_2_-free p-type Si(111)-H substrates via a hydrosilylation reaction using the alkyne group, following our previously reported experimental procedure^31^ (see Figure 1b for monolayer structures). Rad-PTM and αH-PTM were synthesized as previously described.^29^ The three different functionalized surfaces were further characterized by ellipsometry, X-ray photoelectron spectroscopy (XPS), and electrochemistry. Ellipsometry measurements yielded monolayer thicknesses of 16.1 ± 0.5, 21.0 ± 1.0, and 21.8 ± 1.0 Å for Si/EHB, Si/αH-PTM, and Si/rad-PTM, respectively. Such values are in good agreement with the theoretical molecular lengths estimated to be about 14.9, 18.8, and 18.6 Å, respectively (see also Figure S2 in the Supporting Information). XPS analysis of the three modified surfaces revealed characteristic peaks from the Si substrate and from the C 1s and Cl 2p core levels of the attached molecules (Figures S4–S7). For Si/EHB, the high-resolution C 1s spectrum displayed a main component at 285.0 eV corresponding to unresolved contributions of C–C and C=C bonds (Figure S4). Additionally, the Si 2p spectrum did not show any significant oxidation of the underlying silicon surface, in agreement with a dense monolayer (Figure S7). The C 1s and Cl 2p spectra of Si/αH-PTM (Figure S5) and Si/rad-PTM (Figure S6) were comparable. Furthermore, the experimental area under the C–C (C=C and C alpha) and C–Cl peaks was perfectly consistent with the expected 1.1 (15 C–C vs 14 C–Cl bonds) ratio, 1.1 for the αH-PTM, and 1.2 for the rad-PTM, supporting a successful monolayer grafting. Another experimental evidence that the PTM molecules were not altered after grafting was provided by the atomic concentrations of carbon and chlorine determined from the peak areas which were perfectly in agreement with the expected chemical composition, namely 2.2 (αH-PTM) and 2.1 (rad-PTM) against 2.1 (29 carbon vs 14 chlorine atoms). The grafting of sterically hindered bulky PTM introduced some unavoidable oxidation of the underlying silicon surface, as evidenced by the presence of a small peak at a binding energy of 103.0 eV attributable to silicon oxides.^32^ Importantly for the comparison of the electrical properties of the junctions, the Si–O/Si ratio was found to be very similar for both PTM derivatives (Figure S7).

The voltammetric analysis of the three modified surfaces, which was performed under illumination (100 mW cm^–2^, AM 1.5G, SI) to activate the electron conduction of the Si substrate, revealed that only the Si/rad-PTM surface was electroactive (Figure S8). Indeed, the illuminated Si/rad-PTM showed a reversible redox wave corresponding to the PTM(radical)/PTM(anion) process, characterized by a nonideal peak splitting. Such a double peak is believed to be originated from some lattice strain resulting from strong interactions between neighboring PTM units, as previously reported for ferrocenyl monolayers bound to gold.^33^ Such a peak splitting is frequently observed for densely packed electroactive monolayers. Besides, the surface coverage of attached rad-PTM was estimated to be (7.5 ± 0.5) × 10^–11^ mol cm^–2^ from the integration of the anodic peak photocurrent at different scan rates, which is relatively close to the value reported in our previous report, namely (8.5 ± 0.3) × 10^–11^ mol cm^–2^.^31^ Moreover, both anodic and cathodic peak photocurrent densities *j*_pa_ and *j*_pc_ were found to vary linearly with the scan rate (Figure S8b), as expected for a surface-confined reversible redox species.^34^

To obtain further insights into the electronic properties of the modified surfaces, electrochemical impedance spectroscopy (EIS) measurements were performed. More particularly, the flatband potential *V*_fb_ of the silicon surface, i.e., the electrode potential for which there is no space charge region in the semiconductor, was estimated from the commonly used Mott–Schottky plot (*C*^–2^ vs *V*) that gives the space charge capacitance *C*_sc_ as a function of the electrode potential *V* under depletion conditions (i.e., depletion of valence band holes in the space charge region of the p-type surface). The calculated values of *V*_fb_ were in the range 0.26–0.30 V vs the saturated calomel electrode (SCE) and not significantly dependent on the nature of the immobilized molecule (Table S1, Figures S9 and S10). It is noted that these values are very close to the ones extracted from solid-state capacitance measurements (*vide infra*).

### Charge Transport across Metal/Monolayer/Semiconductor (MmS) Junctions

To perform charge transport measurements, the three different functionalized surfaces were soft top-contacted with a fresh EGaIn tip shaped as a cone exhibiting a typical geometrical contact area of about 900–1600 μm^2^. To avoid the influence of photogenerated carriers on the silicon substrate, all the measurements were performed in the dark. Twenty *J–V* traces were recorded applying a reverse bias followed by a forward bias employing a scan speed of 100 mV/s. All junctions were formed and measured with a freshly prepared tip in order to avoid variations in the oxide skin thickness and roughness over time.^35^

**Figure 2:**
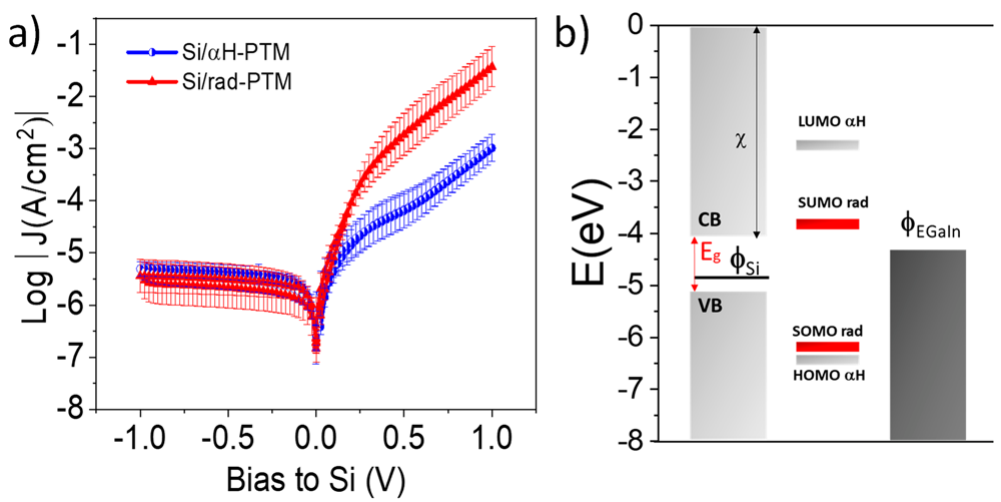
(a) Semilog plot of *J* vs the voltage on Si (V) for Si/PTM//GaOx/EGaIn junctions: rad-PTM (red) and αH-PTM (blue), 20 junctions × 20 traces were measured. (b) Scheme of the band diagram of the interfaces. This representation does not take into account changes in the electron affinity of silicon. *E*_g_ is the band gap energy of Si (1.12 eV),^43^ χ is the silicon electron affinity (4.05 eV),^43^ and φ is the EGaIn work function (4.3 eV).^44^

As shown in Figure 2, Si/αH-PTM and Si/rad-PTM junctions exhibit a clear diode behavior showing good reproducibility as well as a remarkable stability with a 100% yield of the junctions formation (see Table S2 for the statistical data). Other junctions on silicon substrates were already reported to be very reliable, which was attributed to the C–Si bond stability and the smoothness of the silicon surface.^6,7^ As expected for a diode, both junctions show rectification behavior but, remarkably, the Si/rad-PTM//GaOx/EGaIn shows almost two orders of magnitude higher rectification ratio *R*|*J*_1V_/*J*_–1V_| = 10^4.04^ in comparison with the Si/αH-PTM//GaOx/EGaIn with a *R*(|*J*_1V_/*J*_–1V_|) = 10^2.30^, this value being independent of the contact area (Figure S11). Generally, in molecular electronics, the current rectification depends on different factors such as the molecular structure^36,37^ and the band/energy level alignment.^38,39^ In the case of MmS junctions, the molecular conductivity, the Si–C–R dipole, the monolayer quality (i.e., presence of defects), and the monolayer/top contact interface can influence the energy levels alignment which impacts on the charge transport behavior^6,7^ and thus also on the rectification ratio.^40,41^ Although it is well established that the presence of an interfacial dipole layer affects the depletion region (thus the Schottky barrier height), here, similar molecular dipoles are expected for the αH-PTM and the rad-PTM.^42^ Importantly, the distinct rectification ratio is not attributed to the small differences in the content of surface silicon oxide seen by XPS because such a different defect density is expected to have an influence at low applied bias where the Schottky barrier dominates the transport,^32^ which is not the case here. On the contrary, in the high bias regime, where the measured current is dominated by the transport through the monolayer, i.e., when the molecular electronic structure plays a role, we do observe the influence of the molecules. As illustrated in the schematic energy band diagram of Figure 2b, it is clear that the rad-PTM shows a lower SOMO/SUMO band gap compared to the HOMO/LUMO band gap of the αH -PTM. The SOMO/SUMO (−6.24 eV/–3.81 eV) and HOMO/LUMO (−6.40 eV/–2.30 eV) energy values have been determined by DFT calculations (see the Supporting Information), giving values similar to other reported systems.^30^

From the *J–V* plots, it can be seen that at reverse bias the current is negligible for both junctions and practically constant, as expected for an ideal diode behavior in the off state. Remarkably, clear differences are observed at forward bias (i.e., with the diode in the on state). Indeed, the Si/rad-PTM surface displays an enhanced current density (by a factor of 35) in comparison with Si/αH-PTM. As previously mentioned in metal/rad-PTM/metal junctions, such a trend could be attributed to a decrease in the injection barrier due to the participation of the SUMO energy level in the transport.^27,30^ However, in MmS junctions, it must be kept in mind that a second barrier (i.e., a Schottky barrier) arises.

Hence, with the aim to determine the values of the barrier height and to get more insights on the charge transport mechanisms, solid-state impedance spectroscopy measurements (capacitance vs voltage) were carried out. Mott–Schottky analysis was performed to determine the flatband potential |*V*_fb_| which permits to identify different transport regimes. At forward bias, when |*V*_bias_| < |*V*_fb_|, the charge transport is governed by the Schottky barrier, and its magnitude is attenuated by the monolayer. While, when |*V*_bias_| > |*V*_fb_|, the charge transport is governed by the molecule characteristics, and the transport is similar to a metal/molecule/metal (MmM) junction. Thus, this zone allows comparing the charge transport through the molecules without the influence of the Schottky barrier.^7,43^Figure S12 shows the different schematic energy diagrams associated with these different voltages ranges.

### Capacitance–Voltage Measurements and Barrier Height Determination

**Figure 3:**
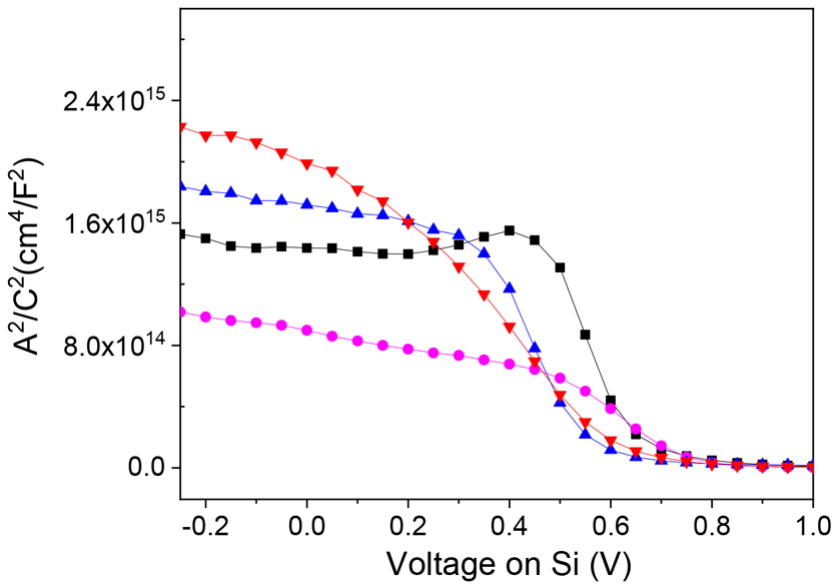
Capacitance–voltage measurements plotted as *A*^2^/*C*^2^ vs bias voltage for Si/layer//GaOx/EGaIn junctions with layer = rad-PTM (red), αH-PTM (blue), SiO_2_ (black) and EHB (pink).

To avoid discharging of the interface states during the determination of the flatband potential, the capacitance was measured at 0.5 MHz and the amplitude of the AC signal was fixed at 50 mV.^6,43,45^ To support our characterization and analysis approach, two additional junctions were examined as reference systems, namely the Si/SiOx//GaOx/EGaIn and the Si/EHB//GaOx/EGaIn (Figures 1 and S17). The EHB layer was chosen since the linker unit (−Ph–C=C–Si) is the same as that in the PTM derivatives, but it is expected to form a more densely packed monolayer due to the absence of the bulky PTM moiety. Figure 3 shows representative Mott–Schottky plots (at 0.5 MHz) for the different interfaces. A typical behavior for a p-type semiconducting substrate is observed with a clear change of the capacitance in the depletion region (∼0–0.75 V). Extracted parameters are average values of three different *C*–*V* measurements (Figures S13–S16).

Table 1 summarizes the different parameters extracted by fitting the data to the Mott–Schottky model (eq S2, Supporting Information). As mentioned earlier, the obtained dopant density values are close to those determined by electrochemical measurements (Figure S10) and with the resistivity range provided by the supplier of the silicon wafers (in the range 5–15 Ω cm). Moreover, considering that the work function of EGaIn/GaO_*x*_ is ca. 4.3 eV vs the vacuum level^46^ and assuming that the potential of SCE is −4.68 eV vs the vacuum energy,^47^ the average *V*_fb_ values extracted from solid-state capacitance measurements can be estimated to be 0.31, 0.37, 0.20, and 0.23 V vs SCE for junctions integrating Si/SiO_*x*_, Si/EHB, Si/αH-PTM, and Si/rad-PTM, respectively. Overall, the values obtained for molecular junctions agree relatively well with those determined from electrochemical measurements in solution.

**Table 1:** Transport Parameters Extracted from the Impedance Measurements of the Different Junctions: Dopant Density (*N*_D_), Flatband Potential |*V*_fb_|, and Barrier Height (ϕ_b_)^a^

junction	*N*_D_ × 10^15^ (cm^–3^)	|*V*_fb_| (V)	ϕ_b_ (eV)
Si/SiOx//GaO_x_/EGaIn	1.4 ± 0.1	0.69 ± 0.02	0.92 ± 0.02
Si/EHB//GaO_x_/EGaIn	5.5 ± 0.5	0.75 ± 0.04	0.94 ± 0.04
Si/αH-PTM//GaO_x_/EGaIn	1.9 ± 0.4	0.58 ± 0.01	0.80 ± 0.01
Si/rad-PTM//GaO_x_/EGaIn	2.5 ± 0.3	0.61 ± 0.03	0.82 ± 0.03

a The values were extracted as an average of three distinct samples.

The interfacial MmS barrier height (ϕ_b_) can be calculated with the above experimentally determined |*V*_fb_| and the *N*_D_ using eqs S5 and S6. The obtained values are given in Table 1. It is noted that the experimental extracted ϕ_b_ value for the Si/SiO_x_//GaO_x_/EGaIn junction agrees quite well with the theoretical calculated value of 0.87 eV (for Si//EGaIn, see Section 5 in the Supporting Information). In order to check the validity of our approach, a complementary extraction method of |*V*_fb_| was done using eq S9, obtaining |*V*_fb_| = 0.61 ± 0.02 V (see the Supporting Information and Figure S15), which is in agreement with the theoretical value of 0.66 V. The PTMs and EHB monolayers have an influence on the depletion region decreasing and increasing the ϕ_b_, respectively. Although the influence of the monolayer quality effect cannot completely be discarded,^7,32^ the observed differences can be associated with the different interfacial dipoles,^6,40,41^ which are expected to be different for the alkyl-terminated layer versus the chlorinated phenyl rings of the PTM moiety.

### *J–V* Curves Analysis

To obtain information about the mechanism governing the PTM-based junctions investigated in this work, we have analyzed in depth the current density–voltage *J–V* curves. At forward bias below the flatband voltage (0 < |*V*_bias_| < |*V*_fb_|), i.e., when most of the bias voltage drops across the semiconductor depletion region, the most likely charge transport mechanisms in this situation are thermionic emission, minority carrier diffusion, and generation recombination.^48,49^ The ideality factor (*n*) and the effective Schottky barrier height (ϕ_eff_), which take into consideration the attenuation caused by the monolayer to the transport, can be obtained by fitting the data to the ideal diode equation (eq S11, see Section 6 in the Supporting Information). Thus, these values are used to determine the mechanism responsible for the charge transport. It is important to mention that eq S11 is valid at low forward bias when the total resistance of the device is negligible.^49,50^

Following this approach, ideality factors of *n* = 1.4 ± 0.1 and *n* = 1.3 ± 0.1 were determined for Si/αH-PTM//GaO_x_/EGaIn and Si/rad-PTM//GaO_x_/EGaIn, respectively (Figure S16). Values of *n* = 1 are expected for ideal thermionic emission. Irregularities within the monolayer could increase the fitted *n* and lower ϕ_eff_(*J–V*). Equal ϕ_eff_ were found for both interfaces: ϕ_eff_ (Si/αH-PTM) = 0.74 ± 0.01 eV and ϕ_eff_(Si/rad-PTM) = 0.74 ± 0.01 eV. The fact of having an ϕ_eff_(*J–V*) < ϕ_b_(*C*–*V*) suggests an inhomogeneous barrier, which could be rationalized by the influence of some monolayer defects.^49,50^

In view of all the relevant values obtained from the experimental data and taking into consideration the similarity between both PTM-based monolayers in terms of intrinsic dipole (almost equal Schottky barrier ϕ_b_), monolayer packing and thickness (similar tunneling distance) and consistent measurement conditions, the only way to rationalize the significant current density enhancement at forward bias in the case of the open-shell interface is the presence of the SOMO/SUMO orbitals. Thus, the energetic proximity of the molecular orbitals with the injection electrode decreases the tunneling barrier leading to an increase of the resulting measured current. An energetic diagram of the interfaces is proposed in Figure 2b. As mentioned above, this phenomenon was already previously observed for MmM interfaces.^27,30^ Remarkably, this interpretation is reinforced when |*V*_bias_| > |*V*_fb_|, i.e., under accumulation, wherein the charge transport is controlled by the tunneling transport through the monolayer^51−53^ and the difference in *J* is more pronounced. In this bias regime, the interface behavior resembles a MmM device.

### Photoresponse Behavior of the Junctions

**Figure 4:**
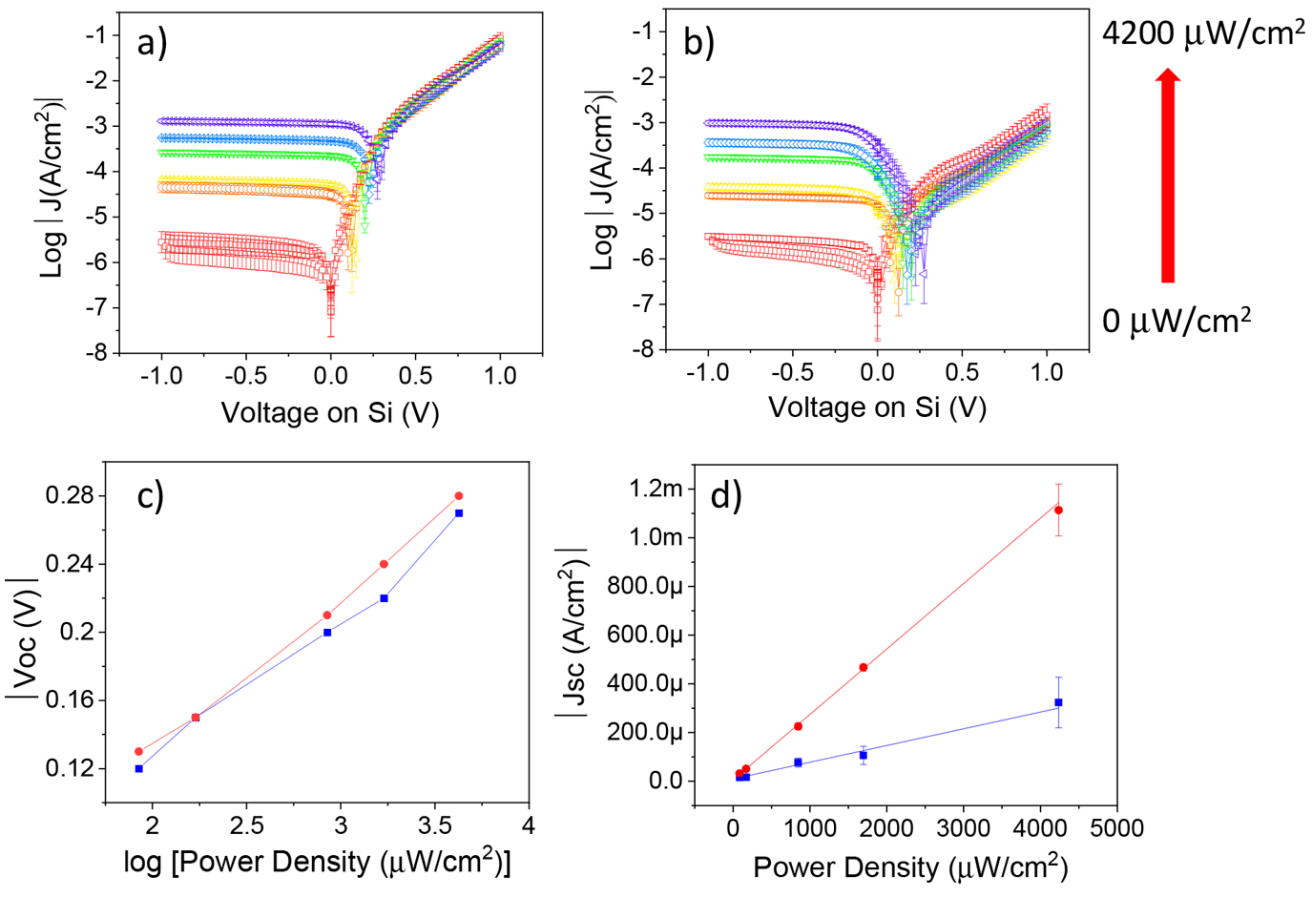
*J–V* measurements under different irradiation powers across (a) Si/rad-PTM and (b) Si/αH-PTM. (c) Open circuit voltage *V*_oc_ and (d) short circuit current density *J*_sc_ of Si/rad-PTM (red) and Si/αH-PTM junctions (blue). The linear regressions using eq S13 gave *S*_ph_ = 68.7 mA/W for Si/αH-PTM and *S*_ph_ = 269.0 mA/W for Si/rad-PTM, with *R*^2^ = 0.96 and 0.99, respectively.

To go a step further with respect to the function utility of the studied systems , the photoresponse of the junctions was inspected. Charge transport measurements under different red laser intensities were performed (λ = 635 nm, photon energy *E*_ph_ = 1.95 eV) which is very close to the PTM radical SUMO–SOMO band gap^30,54^ (see Figure S1 for a scheme of the experimental setup used). In addition, this wavelength does not promote the radical decomposition and is lower than the HOMO/LUMO gap of the αH-PTM. Figures 4a and 4b show the *J–V* curves at different irradiation powers for and Si/αH-PTM//GaO_x_/EGaInSi/rad-PTM//GaO_x_/EgaIn, respectively. Clearly, for both systems, the photocurrent increases as a function of the laser intensity due to the photogenerated charge carriers (see Figure S20 for the band diagram changes upon illumination). The open-circuit voltage |*V*_OC_| (Figure 4c) increases exponentially with the laser power density, which was previously also reported for organic solar cells.^55^ The short-circuit current density |*J*_SC_| increases linearly with the irradiation intensity (Figure 4d), in agreement with the expected typical photodiode characteristics for both junctions.^11,56,57^ Interestingly, the photosensitivity (*S*_ph_) for the rad-PTM is four times higher compared to the Si/αH-PTM, being about 269.0 mA/W against 68.7 mA/W.

These results show an improvement of the photodiode properties by the incorporation of low band gap molecules in the visible spectrum at the interface, as the organic radicals studied here. Our results thus extend the potential use of these systems in photovoltaic devices.^58^

**Figure 5:**
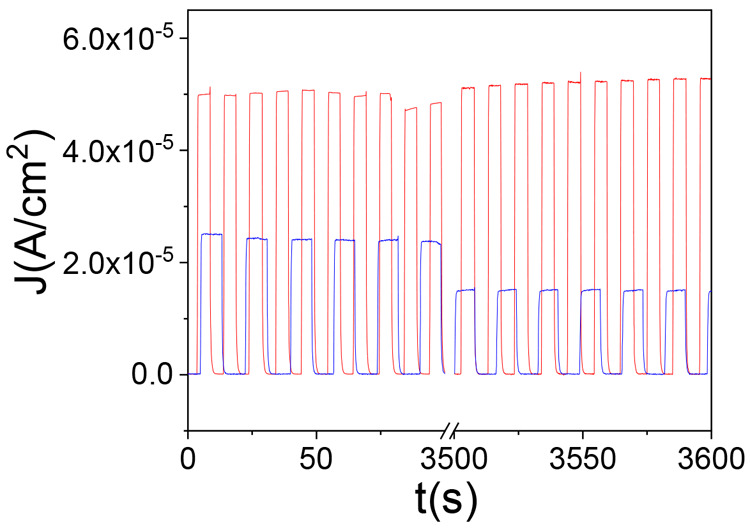
Stability of the junctions under 170 μW/cm^2^ at 0 V bias voltage for 3600 seconds of ON/OFF cycles of irradiation/dark: Si/αH-PTM (blue) and Si/rad-PTM (red).

Finally, another very important aspect is the operational stability. This issue becomes especially relevant for future molecular junctions applications. The ON/OFF ratio values (illumination/dark) for the two monolayers were found to be ∼376 and ∼188 for Si/rad-PTM and Si/αH-PTM, respectively. Charge transport measurements were continuously measured for 1 h, performing pulses of irradiation (6–8 s), under 170 μW/cm^2^ and operating the diode in photovoltaic mode (0 V of applied voltage). As depicted in Figure 5, the Si/rad-PTM-based junction was relatively stable in operation with a negligible loss in the photocurrent over 1 h, whereas an ∼40% loss was observed for the Si/αH-PTM-based junction. Figure S21 shows the full variation of *J* along the 3600 seconds of consecutive cycles performed, showing the photoresponse trend for both monolayers. The origin of such a discrepancy between both interfaces is currently unclear. The difference in the *J*_SC_ magnitude (at 0 V) is in agreement with the above-discussed charge transport measurements in the dark, where the SOMO/SUMO presence in the radical PTM enhances the *J* values.

## Conclusions

In summary, we have demonstrated the successful incorporation of an open-shell molecule into a MmS structure (Si/rad-PTM//GaO_x_/EGaIn), displaying a diode behavior with higher rectification ratio with respect to the closed-shell counterpart. Impedance measurements were performed to analyze in depth the influence of the SOMO/SUMO orbitals on the interface electrical performance. It is demonstrated that the current density in the accumulation regime can be modulated by the incorporation of the radical that has a lower energy gap compared with the nonradical counterpart, improving the energy level alignment. Thus, these results confirm that the modification of Si with functional molecules is an appealing strategy to tune the Si-based junction properties. Additional length dependence studies could give the much-needed understanding on the charge transport mechanism, providing the basis for choosing novel systems with a predesigned junction functionality. In addition, the photoresponse of the PTM-based MmS junctions has been investigated, showing a photosensitivity 4-fold higher for the Si/rad-PTM//GaO_x_/EGaIn in comparison with the closed-shell organic layer. The junctions have shown a remarkable stability for over 3600 seconds of on/off light irradiation consecutive cycles. Our findings can contribute to a long-term vision where such molecular systems could be used in silicon-based devices. In particular, our results can pave the way for the preparation of photoresponsive switches wherein the photodiode behavior can be modulated by the intrinsic properties of the molecular system incorporated into the junction. In addition, the stability of the radicals under irradiation conditions allows for their use in other devices, like MmS solar cells, where the open-shell nature can participate and affect the device performance.

## Supporting Information Available

Additional experimental details, materials, and methods, including schemes of experimental setup; preparation and additional characterization of the monolayers (XPS, cyclic voltammetry, and impedance spectroscopy measurements) and their corresponding junctions (diode junction characteristics and supporting electrical characterization) (PDF)
