# Supplementary material for: Stable Organic Radical for Enhancing Metal–Monolayer–Semiconductor Junction Performance
Source: ACS Appl Mater Interfaces. 2023 Jan 16;15(3):4635–42. doi: 10.1021/acsami.2c15690 (PMC9949700; doi:10.1021/acsami.2c15690)
Supplement: Supplementary file 1 — am2c15690_si_001.pdf [file am2c15690_si_001.pdf]

Supporting Information

Stable Organic Radical for Enhancing Metal-Monolayer-Semiconductor Junctions Performance

J. Alejandro De Sousa,[a,b] R. Pfattner,[a] D. Gutiérrez,[a,†] K. Jutglar,[c] S. T. Bromley,[c,d] J. Veciana,[a] C. Rovira,[a] M. Mas-Torrent,[a] B. Fabre,[e] N. Crivillers[a*]

[a] *Institut de Ciència de Materials de Barcelona (ICMAB, CSIC), Campus de la UAB s/n, Bellaterra, 081093, Spain*

[b] *Laboratorio de Electroquímica, Departamento de Química, Facultad de Ciencias, Universidad de los Andes, 5101 Mérida, Venezuela*

[c] *Departament de Ciència de Materials i Química Física & Institut de Química Teòrica i Computacional (IQTC), Universitat de Barcelona, c/ Martí i Franquès 1-11, 08028 Barcelona, Spain.*

[d] *Institució Catalana de Recerca i Estudis Avançats (ICREA), E-08010 Barcelona, Spain*

[e] *Univ Rennes, CNRS, ISCR (Institut des Sciences Chimiques de Rennes)-UMR 6226, F-35000 Rennes, France.*

† Present address: Leitat Technological Center (LEITAT), Carrer Innovació, 2, 08225 Terrassa, Spain.

Corresponding Author

* Núria Crivillers — Institut de Ciència de Materials de Barcelona, ICMAB-CSIC, Campus UAB, 08193 Bellaterra, Spain. E-mail: ncrivillers@icmab.es

Table of contents

1-Materials S-3

2-Apparatus S-3

2.1-(Photo)electrochemical measurements S-3

2.2-Physico-chemical characterizations of the modified silicon surfaces S-4

2.3-Charge transport measurements through *p*-Si/Molecule/GaO_x/EGaIn junctions S-5

2.3.1 Photoresponse measurements. S-6

2.3.2 Capacitance-voltage measurements. S-6

3-General Procedures S-6

3.1-Preparation of the monolayer-modified p-type Si(111) surfaces S-6

3.2-Surface Coverage Calculation S-8

3.3-Charge transport through the monolayers on Silicon S-9

3.4-DFT calculations S-10

4-Monolayer characterization S-11

4.1-XPS characterization of the monolayers S-11

4.2-Electrochemical characterization of the monolayers S-15

4.2.1. Cyclic voltammetry S-15

4.2.2. Mott-Schottky measurements S-16

4.3-Solid-state capacitance-voltage measurements plotted as A^2/C^2 vs bias voltage S-22

5-Theoretical barrier and flatband potential between silicon and EGaIn S-24

6-Charge transport measurements. S-25

6.1-Schottky barrier height determination for Si/SiO_x S-26

6.2- *J-V* characteristics and charge transport mechanisms S-27

7-Photoresponse characterization S-29

1-Materials

All reagents used were of high quality: tetra-*n*-butylammonium perchlorate Bu_4NClO_4 (Fluka, puriss, electrochemical grade), 1-ethynyl-4-hexylbenzene (Fluorochem, 97%), neutral alumina (from Merck), ultra-pure 18.2 MΩ cm water (Elga Purelab Classic UV, from Veolia), toluene (>99.8%, from Fisher), 1,2-dichlorobenzene (DCB, 98% extra-dry, from Acros), dichloromethane, acetone and ethanol 99.8% (RSE for electronic grade from Carlo Erba). Perchlorotriphenylmethyl radical (rad-PTM) and its precursor perchlorotriphenylmethyl αH (αH-PTM) used in this work were synthesized using the procedure reported by Bejarano and co-workers[1]. The gallium–indium eutectic used to prepare the soft-contact tips were purchased from Sigma-Aldrich.

The chemicals used for cleaning and etching silicon wafer pieces (30% H_2O_2, 96-97% H_2SO_4 and 40% NH_4F solutions) were of VLSI semiconductor grade from Roth (H_2O_2), BASF (H_2SO_4) and Aldrich (NH_4F). 50% HF was of MOS semiconductor grade (Aldrich). All Teflon vials used for cleaning of silicon were previously decontaminated in 3:1 v/v concentrated H_2SO_4/30% H_2O_2 at 100°C for 30 min, followed by copious rinsing with ultra-pure water.

Caution: The concentrated H_2SO_4:H_2O_2 (aq) piranha solution is very dangerous, particularly in contact with organic materials, and should be handled extremely carefully.

2-Apparatus

2.1-(Photo)electrochemical measurements

Cyclic voltammetry measurements were performed with an Autolab electrochemical analyzer (PGSTAT 30 potentiostat/galvanostat from Eco Chemie B.V.) equipped with the GPES and FRA softwares in a home-made three-electrode glass cell. The working electrode, modified Si(111), was pressed against an opening in the cell side using a Teflon circular piece and a FETFE (Aldrich) O-ring seal. An ohmic contact was made on the previously polished rear side of the sample by applying a drop of In-Ga eutectic (Alfa-Aesar, 99.99%). A steel piece was

dropped on the eutectic-coated sample and then the assembly was screwed to the cell using a plastic cap screw. The electrochemically active area of the Si(111) surface (namely 0.38 cm^2) was estimated by measuring the charge under the voltammetric peak corresponding to the ferrocene oxidation on Si(111)-H and compared to that obtained with a 1 cm^2-Pt electrode under the same conditions. For both measurements, the counter electrode was a carbon rod and the system 10^{-2} M Ag^+ | Ag in acetonitrile was used as the reference electrode. All reported potentials are referred to KCl-saturated calomel electrode (uncertainty ± 5 mV) using the reversible ferrocenium/ferrocene couple as the redox probe. Tetra-*n*-butylammonium perchlorate Bu_4NClO_4 was used at 0.1 mol L^{-1} as supporting electrolyte. The electrolytic medium was dried over activated, neutral alumina for 30 min, under stirring and under argon. About 25 mL of this solution was transferred with a syringe into the electrochemical cell prior to experiments. All electrochemical measurements were carried out inside a home-made Faraday cage, at room temperature (20 ± 2 ºC) under constant argon gas flow. The light was provided by a solar simulator with a fluence of 100 mW cm^{-2} (LS0106, LOT Quantum Design) equipped with a AM 1.5G filter. For impedance spectroscopy measurements, the amplitude of the alternating current (ac) signal was 10 mV. For Mott-Schottky experiments, the cell was in the dark and the potential was swept from negative (charge depletion regime) to positive (charge accumulation regime) values at a frequency of 10 kHz (25 mV step potential).

2.2-Physico-chemical characterizations of the modified silicon surfaces

X-ray photoelectron spectroscopy. XPS measurements were performed at room temperature with a SPECS PHOIBOS 150 hemispherical analyzer (SPECS GmbH, Berlin, Germany) in a base pressure of $5x10^{-10}$ mbar using a monochromatic Al Kα radiation (1486.74 eV) as excitation source operated at 300 W. The energy resolution as measured by the FWHM of the Ag 3d5/2 peak for a sputtered silver foil was 0.62 eV. The spectrum was calibrated with respect to the C1s at 284.8 eV.

Spectroscopic ellipsometry. Spectroscopic ellipsometry experiments were performed in the range 300-800 nm at three incidence angles of 65, 70 and 75°, using a Horiba ellipsometer, and analyzed with a two-layer (Si + alkyl) model.

2.3-Charge transport measurements through *p*-Si/Molecule/GaO_x/EGaIn junctions

The "EGaIn measurements" were performed using a home-made measurement set-up (**Figure S1**. The top-electrode was biased and the bottom electrode (p-silicon) was grounded. The *J-V* curves were acquired using a Keithley 2004B. The Keithley was controlled using the software developed with C/C++ and graphical data in Matlab. Back contact was made placing a liquid metal drop on the silicon bottom surface which was scratched with a diamond tip to ensure the Ohmic contact. As we use a p-type semiconductor, EGaIn is negative with respect to the p-Si at forward bias ($V > 0$), while the situation is opposite at reverse bias ($V < 0$).

The measurements were performed under ambient conditions: (T = 21 ± 2°C; RH = 45 ± 10%).

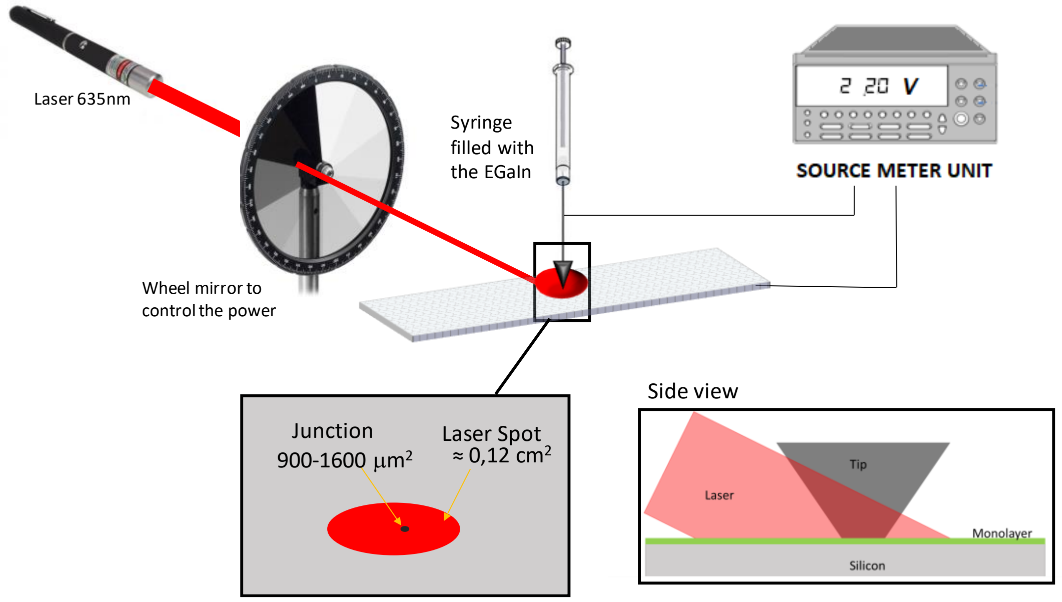

Figure S1. Top: Schematic setup of the home-made system for performing current vs. voltage measurements using the EGaIn as the top electrode. The incident laser was filtered to adjust the desired power intensity. Bottom: Scheme of the laser spot and the tip with their approximate areas and, schematic side view of the junction illumination.

2.3.1 Photoresponse measurements.

Photoelectrical measurements were performed using a Thorlabs laser CPS635, 3R, 635 nm, 4.5 mW, and the power was controlled with a Thorlabs NDC-50C-4M-A - mounted continuously variable ND filter, Ø 50 mm, OD: 0.04-4.0. The intensity of the light was measured with a power meter Thorlabs PM100D coupled with a standard photodiode power sensor (S120C, Si, 400-1100 nm). The spot of the laser was focused on the junction. The alignment between the laser and the junction was kept constant to minimize fluctuations on the incident light intensity. The pulses were performed using a homemade setup with a motor connected to a rotating shutter adequate for doing the intermittent irradiation without switching on and off the incident laser.

2.3.2 Capacitance-voltage measurements.

The capacitance C was determined using a Novocontrol Alpha-A coupled with an electrochemical module POT-GAL. The equipment was controlled with winDETA 5.85 software. During the experiments, the set-up was maintained in darkness and the dc-voltage was swept biasing the junctions first in reverse followed by forward mode. The capacitance was measured at a frequency of 0.5 MHz with the amplitude of the alternating current (ac) set to 50 mV.

3-General Procedures

3.1-Preparation of the monolayer-modified p-type Si(111) surfaces

All single side polished Si(111) samples (*p*-type, boron doped, 5-15 Ω cm, thickness = 525 ± 25 μm, from Sil'tronix) were cut into 1.5 × ~4.0 cm^2 pieces from the same silicon wafer to ensure the maximum reproducibility of hydrogen-terminated and further molecular monolayer-modified surfaces. The sample was sonicated for 10 min successively in acetone (RSE for electronic grade, Carlo Erba), ethanol (99.8%, RSE for electronic grade, Carlo Erba) and ultra-pure 18.2 MΩ cm water (Elga Purelab Classic UV, Veolia). It was then cleaned in 3:1 v/v concentrated H_2SO_4/30% H_2O_2 at 100°C for 30 min, followed by copious rinsing with ultra-pure water.

The surface was first etched in aqueous HF 5-10% for 1 min, then with argon-degassed ppb grade 40% aqueous NH_4F for 20 min at room temperature. The NH_4F solution was thoroughly degassed with argon for at least 1 h prior to the immersion of the piranha-treated surface. After etching, the Si-H sample was rinsed with argon-saturated water, blown dry with argon, and transferred immediately into a Pyrex Schlenk tube containing the deoxygenated alkyne derivative: 1-ethynyl-4-hexylbenzene (Fluorochem, 97%), **αH-PTM** or **rad-PTM**, at ca. 7-8 mM in 1,2-dichlorobenzene (DCB, 98% extra-dry, from Acros). An aluminum foil was systematically put around the glassware to avoid the possible photochemical degradation of the alkyne derivative. The hydrosilylation reaction was thermally activated at 145°C for 20 h and the alkyne solution was kept under a pressure of argon during the reaction. After reaction, the monolayer-modified silicon surface was thoroughly rinsed with toluene (>99.8%, from Fisher) and dichloromethane (RSE for electronic grade, Carlo Erba), then dried under argon. The modified surfaces prepared from 1-ethynyl-4-hexylbenzene, **αH-PTM** and **rad-PTM** were denoted as **Si/EHB**, **Si/αH-PTM** and **Si/rad-PTM**, respectively.

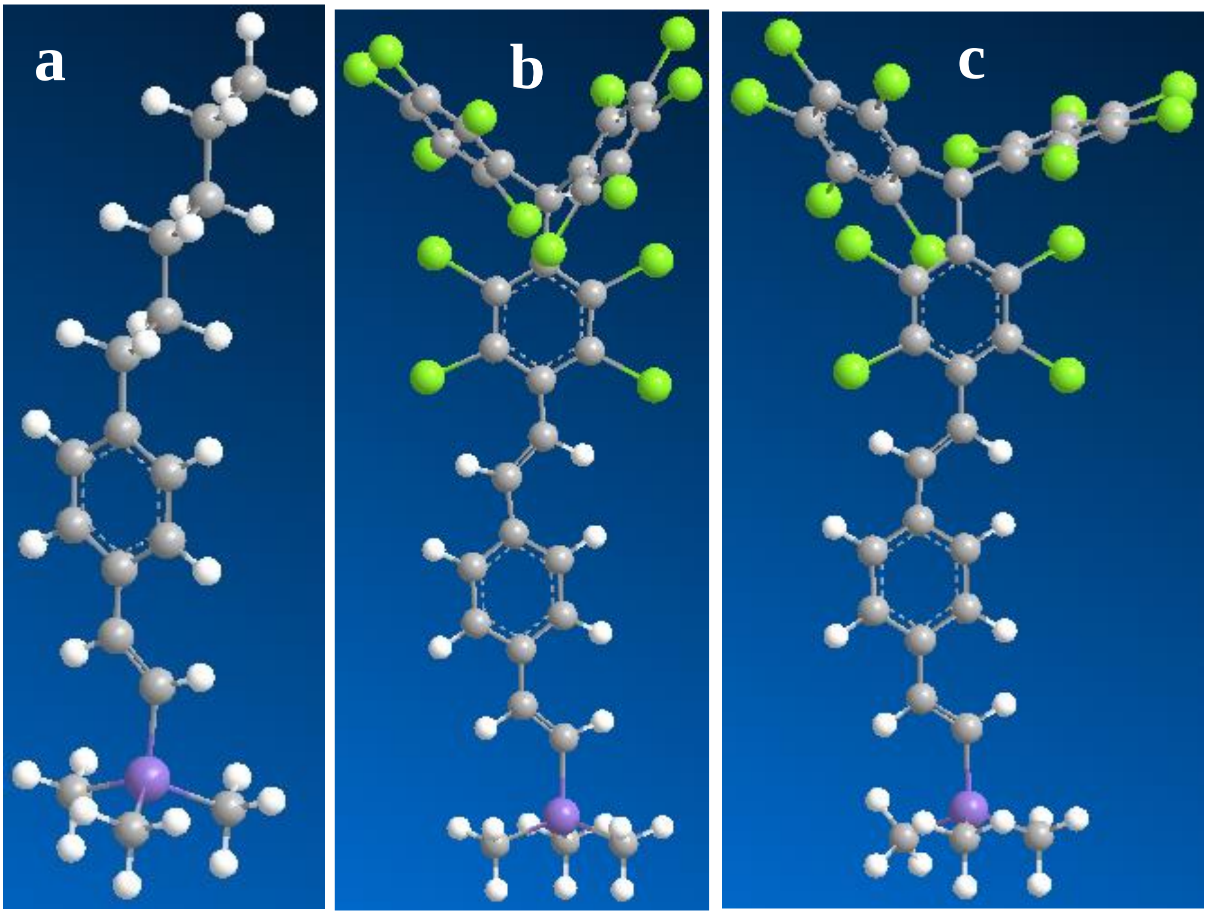

Figure S2. Chemical structures of **Si/EHB** (a), **Si/αH-PTM** (b) and **Si/rad-PTM** (c) from ChemDraw. The surface is represented by the $Si(CH_3)_3$ group.

3.2-Surface Coverage Calculation

The surface coverage was calculated from the **Equation S1**[2,3]:

$$\Gamma = \frac{A_{peak}}{nFSv} \qquad \textbf{Equation S1}$$

where Γ is the surface coverage in mol cm^{-2}, A_{peak} is the integrated area of the anodic or cathodic voltammetry peak, n is the number of electrons transferred (in this case $n = 1$), F is Faraday's constant, S is the electrode surface area and v is the potential scan rate.

3.3-Charge transport through the monolayers on Silicon

The modified *p*-Si substrates were top-contacted with the EGaIn/GaOx tips. The tips were prepared following reported procedures.[4,5] In particular, for this work, for the EGaIn tip shaping was done as follows: A 10 µL Hamilton syringe is filled with 5 µL of Eutectic GaIn alloy, then the syringe is held with a ThorLab support (KM100V) and its movement is controlled by a 3-axis traveller. Then, to fabricate the EGaIn cone with the desired radius, an EGaIn drop is carefully placed on a clean gold substrate and then the syringe is retracted carefully using the 3-axis traveller. Syringe and platform are moved upwards and downwards simultaneously in opposite directions to shape the cone. The tip geometrical contact area was about 900-1,600 μm^2. *J-V* traces were recorded applying a reverse bias followed by a forward bias employing a scan speed of v = 100 mV/s. For each sample, several *J-V* curves were collected at 100 mV/s. The tip was freshly prepared for each junction to avoid changes in the oxide layer thickness and roughness. For the photovoltaic measurements, 15 curves (3 junctions x 5 curves) were recorded.

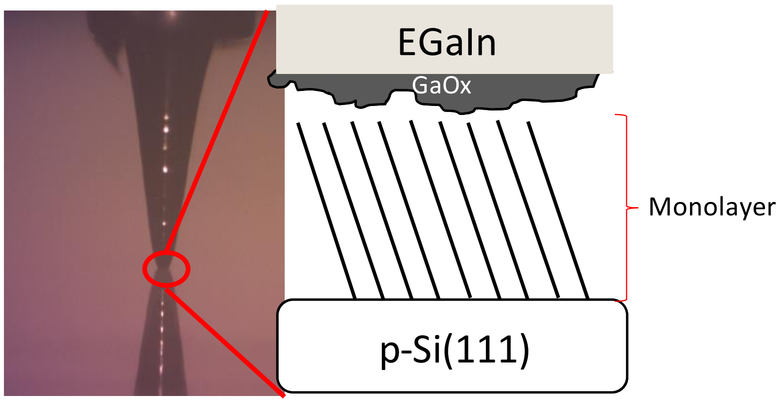

Figure S3. Picture of the junction (left) using EGaIn/GaOx as the top-electrode to contact the organic monolayer covalently bound to p-Si(111) (schematized on the right), used as the bottom electrode.

3.4-DFT calculations

The optimized geometrical structures and molecular orbital energies of **Si/αH-PTM** and **Si/rad-PTM** were computed using density functional theory (DFT) based calculations as implemented in the FHI-AIMS package.[6] The surface-grafted molecules were represented by –$Si(CH_3)_3$ terminated models (see **Figure S2**). All DFT calculations employed the hybrid PBE0[7] exchange-correlation functional which has been shown to be suitable for calculating the electronic properties of PTM-type radicals.[8] A "light/Tier 1" all-electron atom-centered numerical basis set was used throughout, and non-bonded dispersive van der Waals interactions were taken into account using the Tkatchenko-Scheffler approach.[9]

4-Monolayer characterization

4.1-XPS characterization of the monolayers

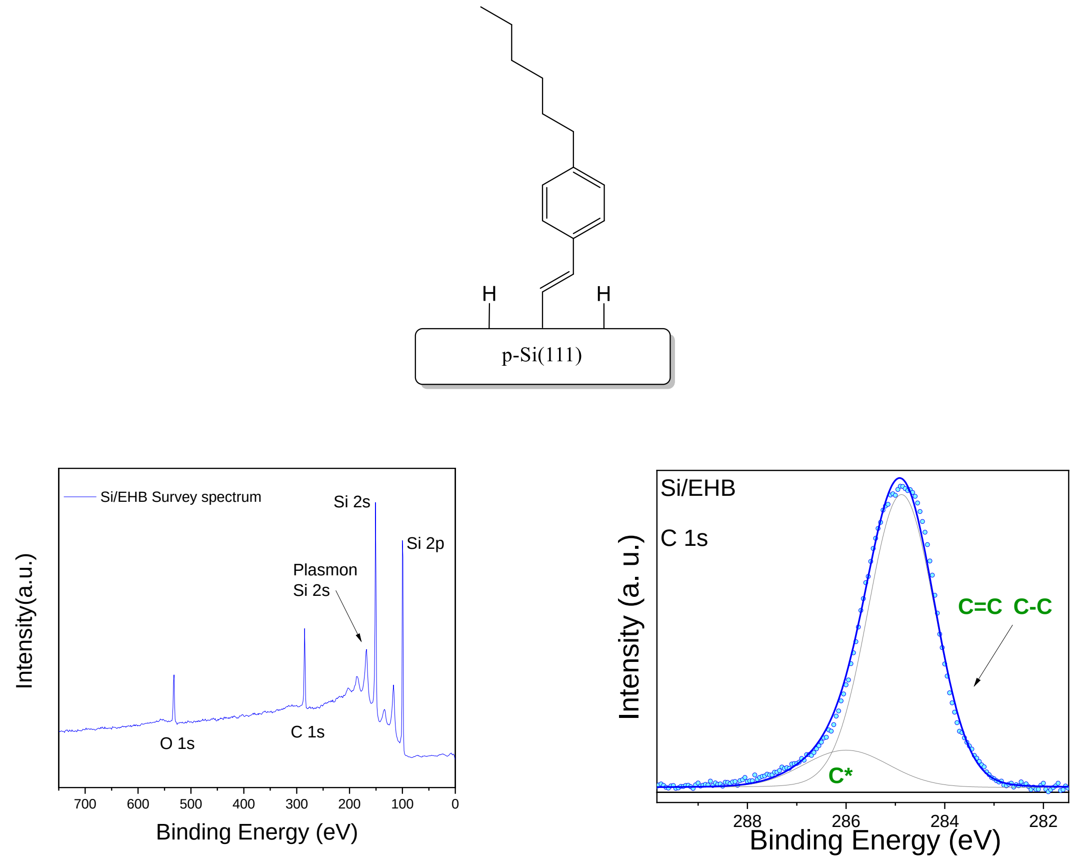

Figure S4. Survey and high-resolution C 1s spectra of **Si/EHB**. The C 1s spectrum shows a main component at 285.0 eV corresponding to unresolved contributions of C-C and C=C bonds. The other component at 285.8 eV may indicate the existence of adventitious carbon (C*, such as C-O bonds) or the contribution of small asymmetry in the main peak.[10–12]

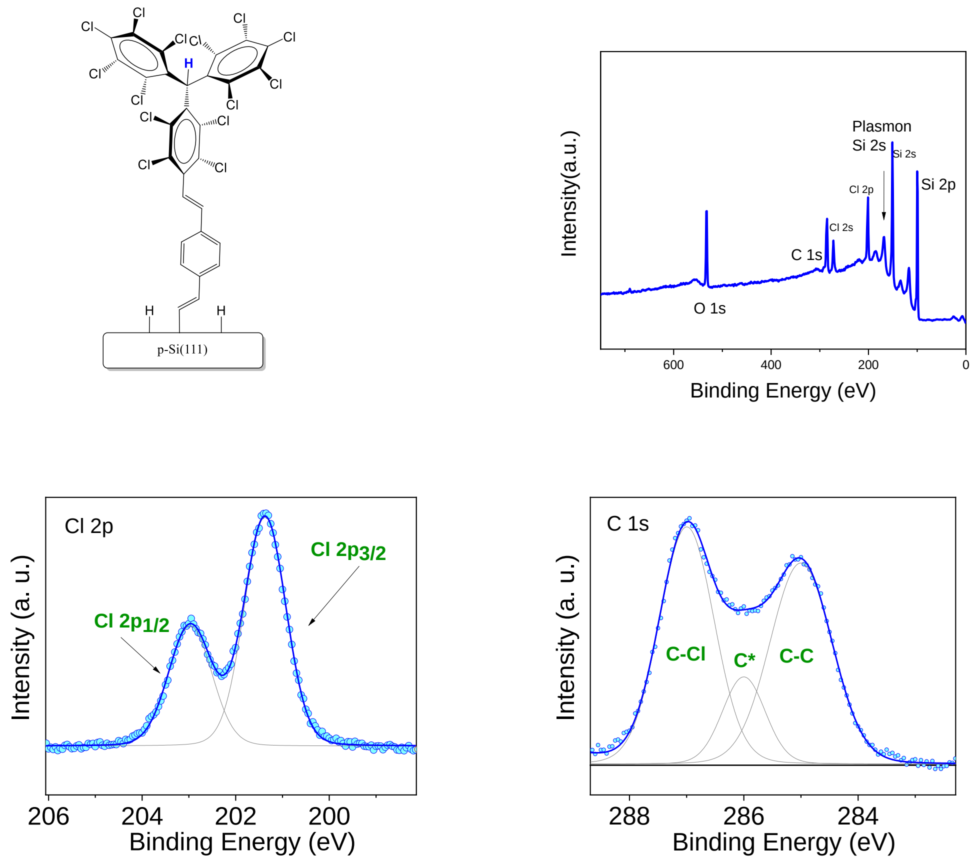

Figure S5. Survey (top) and high-resolution C 1s (bottom right) and Cl 2p (bottom left) of **Si/αH-PTM**. The C 1s spectrum shows two main peaks at 285.0 and 287.0 eV assigned to C-C (C=C and C alpha) and C-Cl bonds, respectively. The experimental ratio between the areas under the C-C and C-Cl peaks is estimated at 1.1, in agreement with the theoretical ratio of 1.1 (15 C-C vs 14 C-Cl bonds). A third component at 286.0 eV can be fitted indicating the presence of some adventitious carbon (C*, such as C-O bonds). The Cl 2p spectrum displays a typical doublet with both components at 201.4 ($2p_{3/2}$) and 203.0 ($2p_{1/2}$) eV, attributed to the chlorinated phenyl rings of PTM.

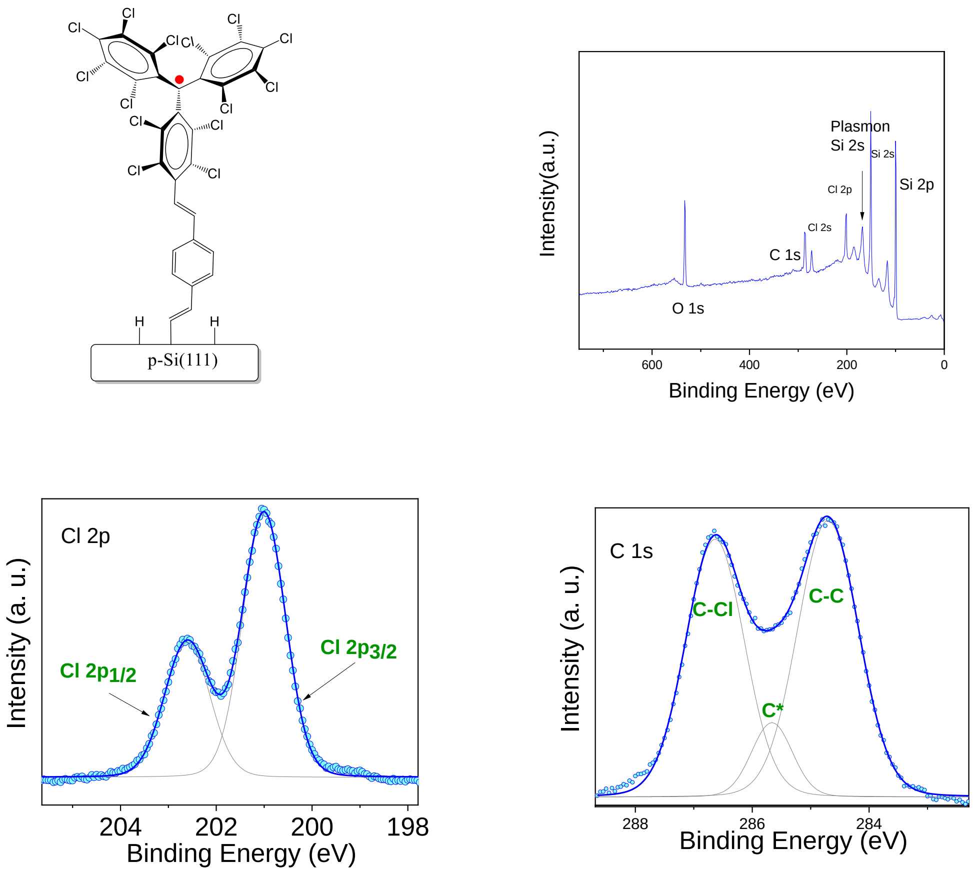

Figure S6. Survey (top) and high-resolution C 1s (bottom right) and Cl 2p (bottom left) spectra of **Si/rad-PTM**. The C 1s spectrum shows two main components at 284.7 and 286.6 eV assigned to C-C (C=C and C alpha) and C-Cl bonds, respectively. The experimental ratio between the areas under the C-C and C-Cl peaks is estimated at 1.2 in close agreement with the theoretical ratio of 1.1 (15 C-C vs 14 C-Cl bonds). A third component at 285.7 eV is visible indicating the presence of some adventitious carbon (C*, such as C-O bonds). The Cl 2p spectrum displays a typical doublet with both components at 201.0 ($2p_{3/2}$) and 202.6 ($2p_{1/2}$) eV, attributed to the chlorinated phenyl rings of PTM.

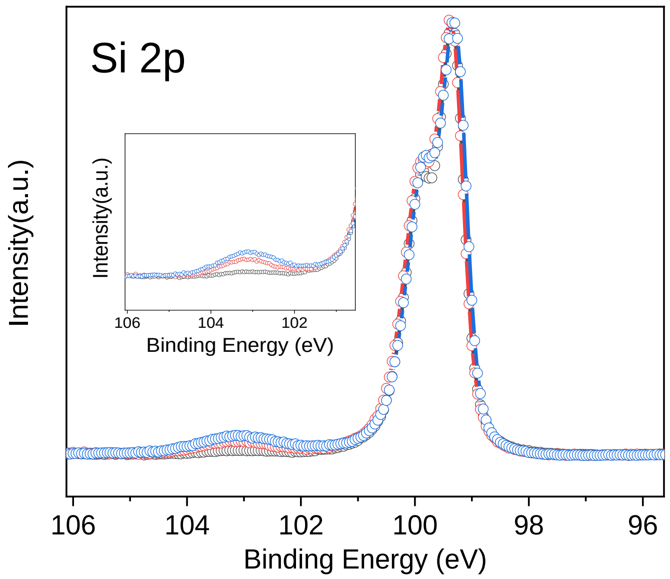

Figure S7. High-resolution Si 2p XPS spectra for the **Si/EHB** (black), **Si/αH-PTM** (red), **Si/rad-PTM** (blue). The Si 2p spectrum shows a main peak at 99.9 eV and 99.4 eV corresponding to Si $2p_{1/2}$ and Si $2p_{3/2}$ components, respectively. The Si 2p spectrum shows the additional presence of a broad and low intense peak at ca. 103.0 eV attributable to silicon oxide species formed from the unavoidable oxidation of a certain content of Si-H sites remaining after the grafting of the sterically hindered PTM.

4.2-Electrochemical characterization of the monolayers

4.2.1. Cyclic voltammetry

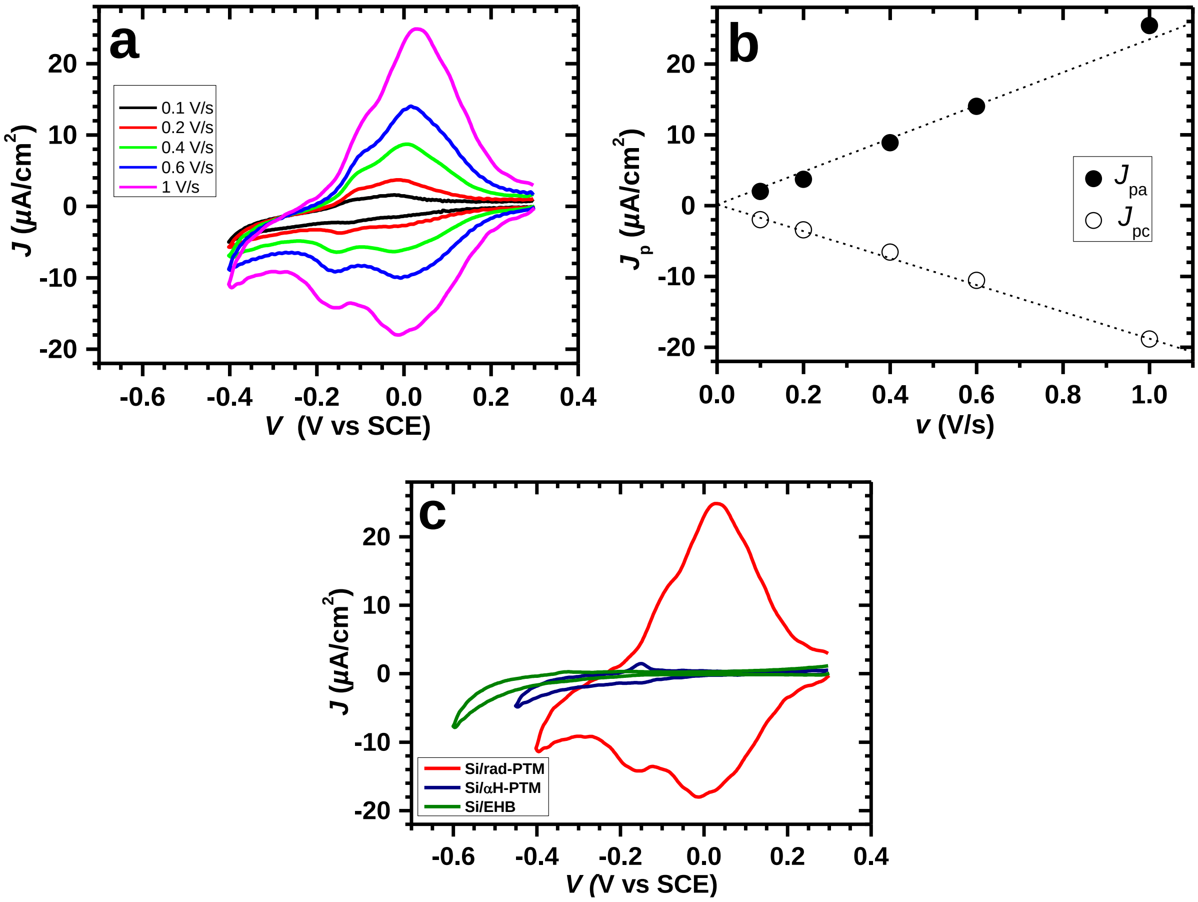

Figure S8. a) Cyclic voltammograms (CVs) under simulated sunlight (100 mW cm^{-2}; AM 1.5G) of **Si/rad-PTM** in CH_3CN + 0.1 M Bu_4NClO_4. b) Plots of anodic (J_{pa}) and cathodic (J_{pc}) peak photocurrent densities corresponding to the redox couple PTM(radical)/PTM(anion) vs. the potential scan rate *v*. c) Comparative CVs at 1 V/s of **Si/rad-PTM**, **Si/αH-PTM** and **Si/EHB** under simulated sunlight.

4.2.2. Mott-Schottky measurements

The flatband potential of each modified surface has been determined from commonly used Mott-Schottky plots ($\frac{A^2}{C^2} - V$, **Equation S2**) where *C* was calculated using the equivalent electrical circuit shown in **Figure S9**.

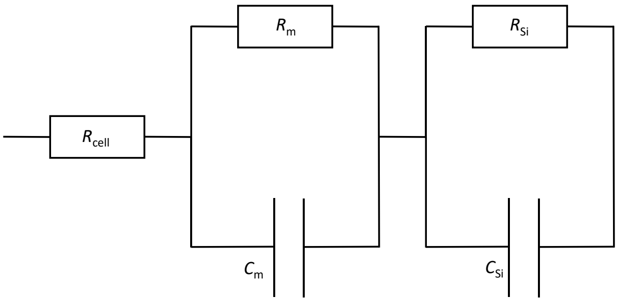

Figure S9. Equivalent electrical circuit used to calculate *C* where R_{cell} is the cell resistance, R_m and C_m are the monolayer resistance and capacitance, respectively, and R_{Si} and C_{Si} are the silicon resistance and capacitance, respectively.

$$\frac{A^2}{C^2} = \frac{2}{qN_D\varepsilon_0\varepsilon_r}\left(V - V_{fb} - \frac{kT}{q}\right) \qquad \textbf{Equation S2}$$

where:

- *C* is the depletion capacitance;
- N_D is the the dopant density;
- V_{fb} is the flatband potential
- *q* is the electron charge ($1.602 \times\cdot 10^{-19}$ C);
- ε_r is the relative permittivity of Si (11.7);
- ε_0 is the vacuum permittivity ($8.85 \times\cdot 10^{-12}$ F m^{-1});
- *A* is the electrode surface area;
- *V* is the applied potential;

- k is the Boltzmann constant ($1.38 \cdot \times 10^{-23}$ J K^{-1});

- T is temperature

N_D can be determined from the slope (b) of the linear part of the $\frac{A^2}{C^2}$-V plot

$$N_D = \frac{2}{q\varepsilon_0\varepsilon_r b}$$ **Equation S3**

and V_{fb} is given by

$$V_{fb} = -\frac{a}{b} - \frac{kT}{q}$$ **Equation S4**

where a is the intercept of the linear part.

Linear $\frac{A^2}{C^2}$ - V plots are obtained in a potential window ranging from -0.80 to -0.40 V, -0.60 to -0.20 V and -0.70 to -0.20 V vs Saturated Calomel Electrode (SCE) for **Si/rad-PTM**, **Si/αH-PTM** and **Si/EHB**, respectively, the slope and the intercept of which enable the flatband potential and the dopant density to be determined (**Figure S10**). The N_D values obtained for the three surfaces are relatively in good agreement with the resistivity value given by the wafer manufacturer (5-15 ohm cm).

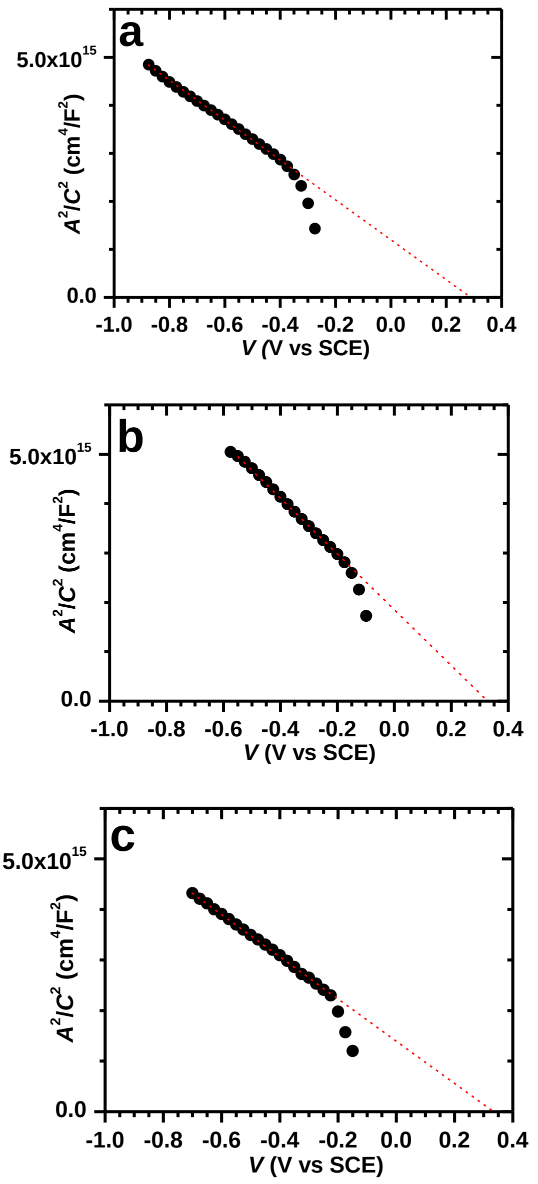

Figure S10. Mott-Schottky C^{-2} $A^2 - V$ plots in the dark at 10 kHz of a) **Si/rad-PTM** (N_D = 3.1 x 10^{15} cm^{-3}, i.e. 4.2 ohm cm, and V_{fb} = 0.26 V vs SCE), b) **Si/αH-PTM** (N_D = 2.15 x 10^{15} cm^{-3}, i.e. 6.0 ohm cm, and V_{fb} = 0.30 V vs SCE) and c) **Si/EHB** (N_D = 2.87 x 10^{15} cm^{-3}, i.e. 4.5 ohm cm, and V_{fb} = 0.30 V vs SCE) in CH_3CN + 0.1 M Bu_4NClO_4.

Table S1. Monolayer Thickness and Flatband Potential (V_{fb}) for the three investigated modified *p*-Type Si(111) surfaces. The values are given versus Saturated Calomel Electrode (SCE).

Surface	Ellipsometric thickness / Å	V_{fb} (V) vs SCE
**Si/EHB**	16.1 ± 0.5	0.30 ± 0.05
**Si/αH-PTM**	21.0 ± 1.0	0.30 ± 0.05
**Si/rad-PTM**	21.8 ± 1.0	0.26 ± 0.05

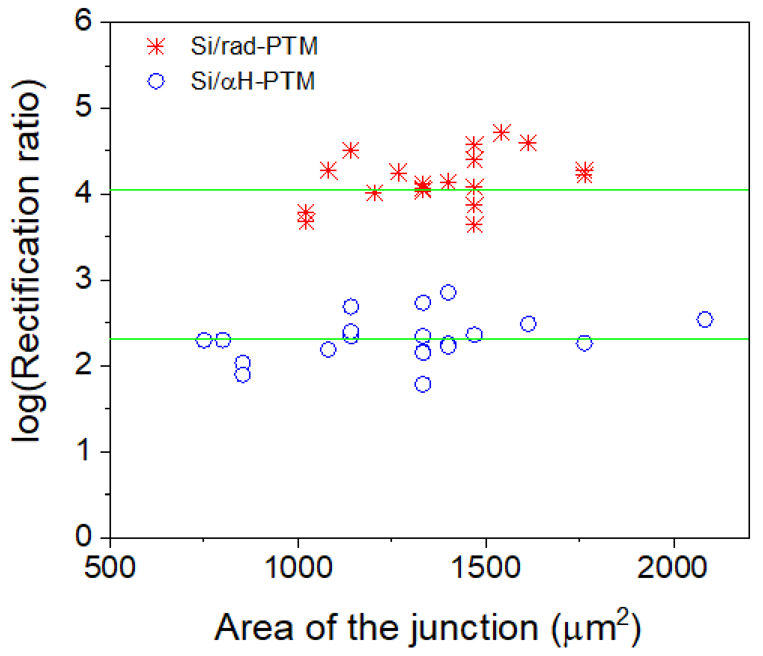

Figure S11. Plot of the log *R* (|J_{1V}/J_{-1V}|) versus the contact area between the EGaIn electrode and the α-H and radical PTM monolayers.

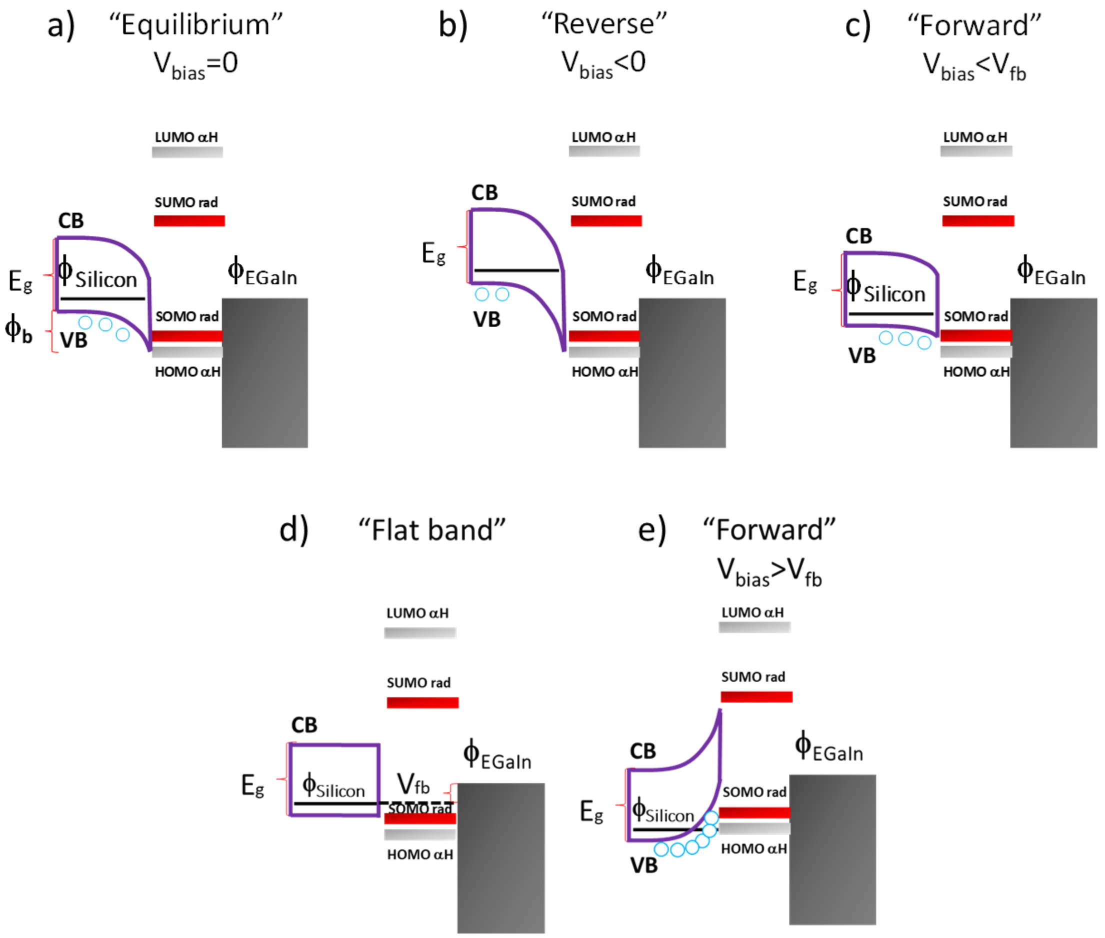

Figure S12. Schematic energy diagrams for Si/PTM//GaOx/EgaIn at different situations of applied bias in the dark.

Figure S12-a shows that upon the equilibration of the Fermi energies, the Schottky barrier is generated (*ϕ_b)*. This can be seen as a potential barrier that avoids the carriers' continuous flow. At reverse bias ($V_{bias}<0$, e.g. -1 V) this barrier increases (**Figure S12-b**), and the transport of the majority carriers is limited. Therefore, the current is negligible and practically constant, indicating a close-to-ideal diode behavior. The magnitude of the current in this voltage range is related to the minority carrier diffusion or thermally generation-recombination process and, it is a function of the Schottky barrier. At forward bias (V_{bias}>0) and when $V_{bias}<V_{fb}$ (**Figure S12-c),** the Schottky barrier governs the charge transport. Thermionic emission is the most common mechanism to describe the *J-V* response when the applied bias falls over the space charge region (semiconductor transport control). Thus, the bias applied decreases the barrier and the majority

carriers start to flow by thermionic emission. At some point, the applied bias eliminates the semiconductor band bending (**Figure S12-d**). This V_{bias} is the so-called flatband voltage (V_{fb}). For $V_{bias}>V_{fb}$, even at +1 V, there is not barrier imposed by the semiconductor (**Figure S12-e**), and the charge transport is governed by the molecule characteristics resembling a Metal/molecule/Metal junction. Thus, in this voltage range, the comparison of the charge transport through the molecules without the influence of the Schottky barrier can be done. Based on this and taking into consideration previous works with similar molecules where we demonstrated a clear influence of the rad-PTM for which the SOMO/SUMO band gap is lower compared to the HOMO/LUMO band gap of the αH –PTM, we hypothesize that in this zone the transport mechanism is tunneling, assisted by the frontier orbitals of the radical.

4.3-Solid-state capacitance-voltage measurements plotted as A^2/C^2 vs bias voltage

Mott-Schottky relationship (**Equation S2**) was used to determine the flatband potential and the dopant density for the different interfaces.

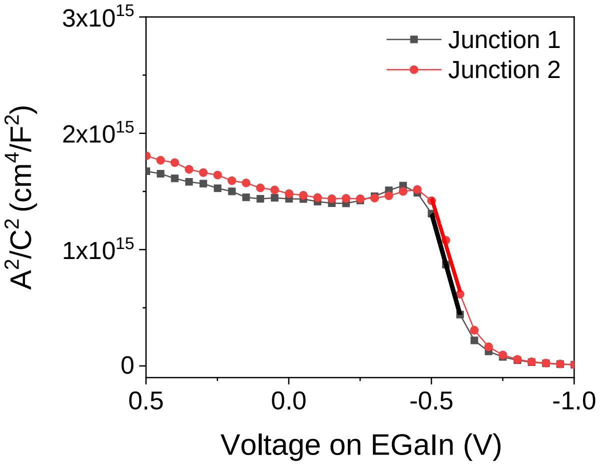

Figure S13. Mott-Schottky plots from the impedance measurements of two junctions **Si/SiO_x**//GaOx/EGaIn at 0.5 MHz. For y = bx + a: a (in cm^4 F^{-2}) = {(a1) 5.66 x 10^{15}; (a2) 5.47 x 10^{15}} and b (in cm^4 F^{-2} V^{-1}) ={(b1) 8.70 x 10^{15}; (b2) 8.05 x 10^{15}}.

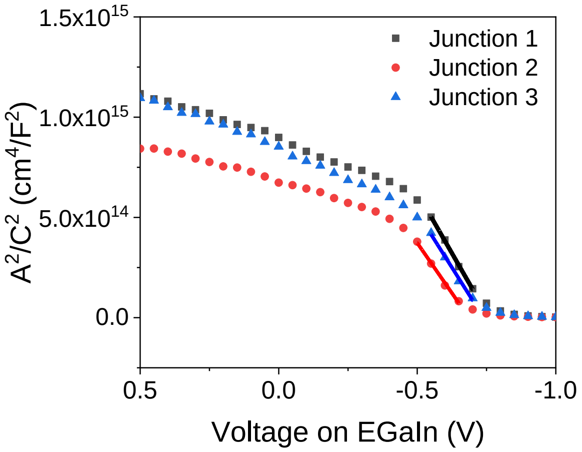

Figure S14. Mott-Schottky plots from the impedance measurements of three junctions **Si/EHB** //GaOx/EGaIn at 0.5 MHz. For y = bx + a: a (in cm^4 F^{-2})={(a1) 1.82 x 10^{15}; (a2) 1.37 x 10^{15}; (a3) 1.62 x 10^{15}} and b (in cm^4 F^{-2} V^{-1}) ={(b1) 2.40 x 10^{15}; (b2) 2.00 x 10^{15}; (b3) 2.19 x 10^{15}}.

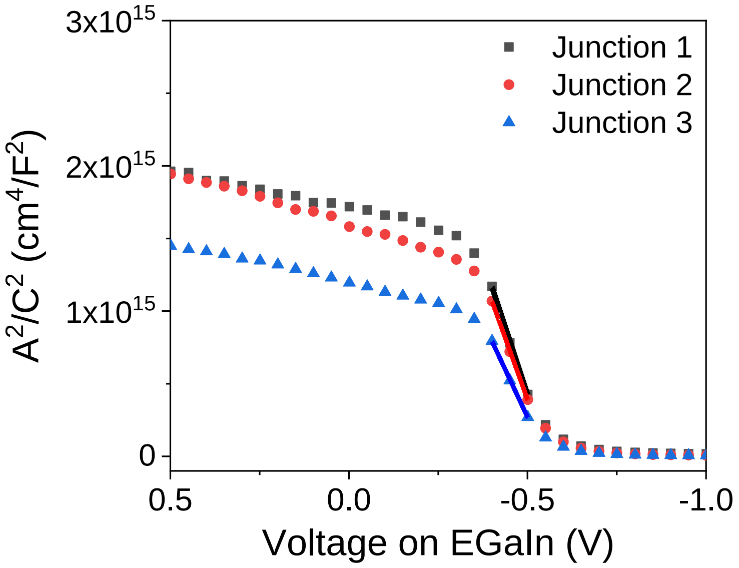

Figure S15. Mott-Schottky plots from the impedance measurements of three junctions **Si/αH-PTM**//GaOx/EGaIn at 0.5 MHz. For y = bx + a: a (in cm^4 F^{-2})={(a1) 4.15 x 10^{15}; (a2) 3.79 x 10^{15}; (a3) 2.90 x 10^{15}} and b (in cm^4 F^{-2} V^{-1})={(b1) 7.45 x 10^{15}; (b2) 6.80 x 10^{15}; (b3) 5.25 x 10^{15}}.

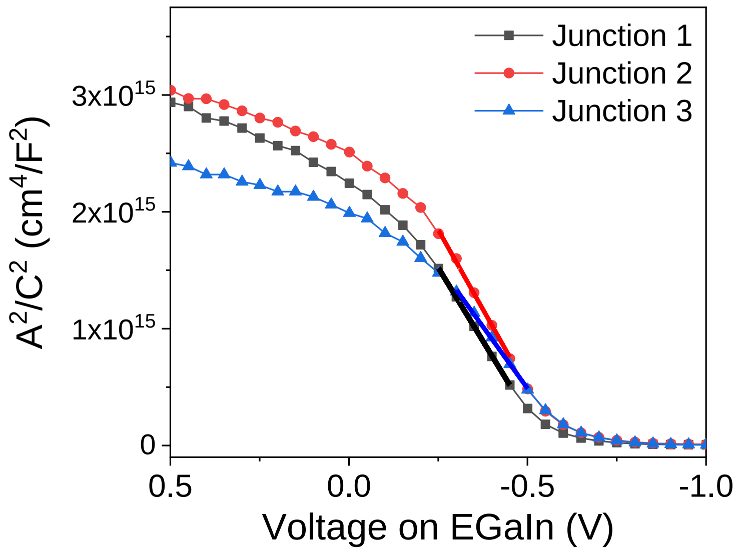

Figure S16. Mott-Schottky plots from the impedance measurements of three junctions **Si/rad-PTM**//GaOx/EGaIn at 0.5 MHz. For y = bx + a: a (in cm^4 F^{-2})={(a1) 2.77 x 10^{15}; (a2) 3.20 x 10^{15}; (a3) 2.61 x 10^{15}} and b (in cm^4 F^{-2} V^{-1}) ={(b1) 5.01 x 10^{15}; (b2) 5.43 x 10^{15}; (b3) 4.24 x 10^{15}}.

For a *p*-Silicon//Metal junction:

Knowing V_{fb} and the dopant density, it is possible to calculate the Schottky barrier height ϕ_b for a MmS interface following the **Equations S5 and S6**.

$$\emptyset_b = -qV_{fb} + q\xi_{VB} \quad \textbf{Equation S5}$$

$$\xi_{VB} = \frac{KT}{q} ln\left(\frac{Nv}{N_D}\right) \quad \textbf{Equation S6}$$

Where ϕ_b is the Schottky barrier height, V_{fb} is the flatband potential, Nv is the number of effective states in the valence band (1.04 x 10^{19} cm^{-3} for silicon),N_D is the acceptor (dopant) concentration (extracted by **Equation S2**), q is the electron charge (1.602 ×·10^{-19} C), k is the Boltzmann constant (1.38 × 10^{-23} $J.K^{-1}$) and T is temperature.

5-Theoretical barrier and flatband potential between silicon and EGaIn

$$\emptyset_b = (E_G + \chi - \varphi) \quad \textbf{Equation S7}$$

Using the **Equations S5 and S6**, we can write

$$qV_{fb} = kT\ ln\left(\frac{Nv}{N_D}\right) - \emptyset_b \quad \textbf{Equation S8}$$

Where:

- $\emptyset_b$ is the Schottky barrier height; V_{fb} is the flatband potential; E_G is the band gap energy of Si (1.12 eV);[13] χ is the silicon electron affinity (4.05 eV)[13]; φ is the EGaIn work function (4.3 eV)[14]; Nv is the number of effective states in the valence band (1.04 x 10^{19} cm^{-3} for silicon); N_D is the dopant density (taken as the average from the values determined from the Mott-Schottky analysis, i.e. 2.84 x 10^{15} cm^{-3}).

Using these values in the **Equations S7 and S8**, we obtain:

$$\phi_{\mathrm{b}} = 0.87\ eV$$

$$V_{fb} = -0.66\ V$$

$$|V_{fb}| = 0.66\ V$$

6-Charge transport measurements.

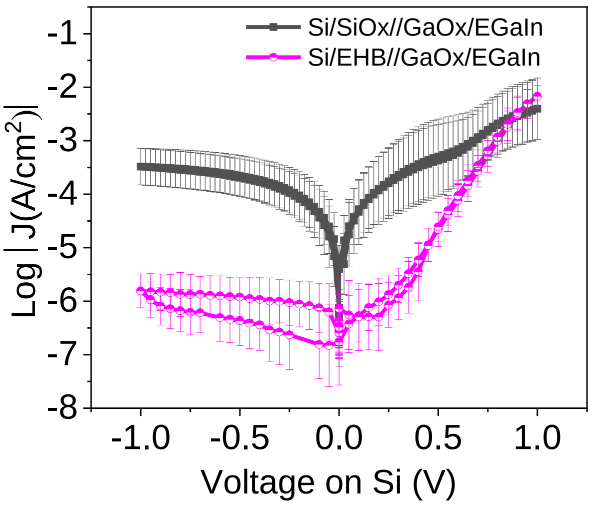

Figure S17. Charge transport measurements across the **Si/layer**//GaOx/EGaIn junctions acquired under ambient conditions.

Table S2. Statistics of electrical characterizations of the four surfaces in the dark.

Surface	Number of Junctions.	Number of curves per junction.	Total Number of curves.	Yield%
Si/SiO$_x$	6	20	120	100
Si/EHB	8	20	160	100
Si/αH-PTM	20	20	400	100
Si/rad-PTM	20	20	400	100

6.1-Schottky barrier height determination for Si/SiO$_x$

Thermally grown SiO_2 is an insulator with a wide bandgap (E_g = 8.9 eV) and a high resistivity of about $\rho = 10^{17}\ \Omega\cdot cm$.[15] Oxidation of Si wafers under ambient conditions leads to a thin layer (d_{SiOx} = 0.6 nm) of SiO_x (with 1 < x < 2) which shows a much lower resistivity compared to SiO_2.[16] Thus, when contacting *p*-type silicon substrates with the top contact EGaIn, the thin native SiO_x can be assumed to work as a resistor connected in series with the Schottky junction (*i.e. p*-type Si/EGaIn junction). This assumption is plausible, because the resistivity of GaO_x is much lower compared to SiO_x, and most of the voltage drops on the native oxide. An ideal Schottky diode of this type can be described by the following expression:

$$I = I_0 \cdot [\exp(q(V_D - RI)/nkT - 1)] \qquad \textbf{Equation S9}$$

… with I the forward current, V_D the forward bias voltage, R the resistance of the native oxide layer.

This equation does not have an analytical solution for R, but plotting the current density of the junction versus forward bias allows extracting V_{fb} and R_{SiOx} by linear regression. In other words, this means that at bias voltages $V_D < V_{fb}$, the diode current is limited by the Schottky barrier, while at a bias voltage $V_D > V_{fb}$ the current is limited by the resistor in series (SiO_x) in sofar as there is no Schottky barrier. This allows extracting the V_{fb} with a value of about $|V_{fb}|$ = 0.61 ±

0.02 V. The resistivity of SiO_x was estimated to be about $\rho_{SiOx} = (8 - 11) \times 10^{10}$ Ω·cm which is much lower than the one expected for SiO_2 and in good agreement with literature.[16]

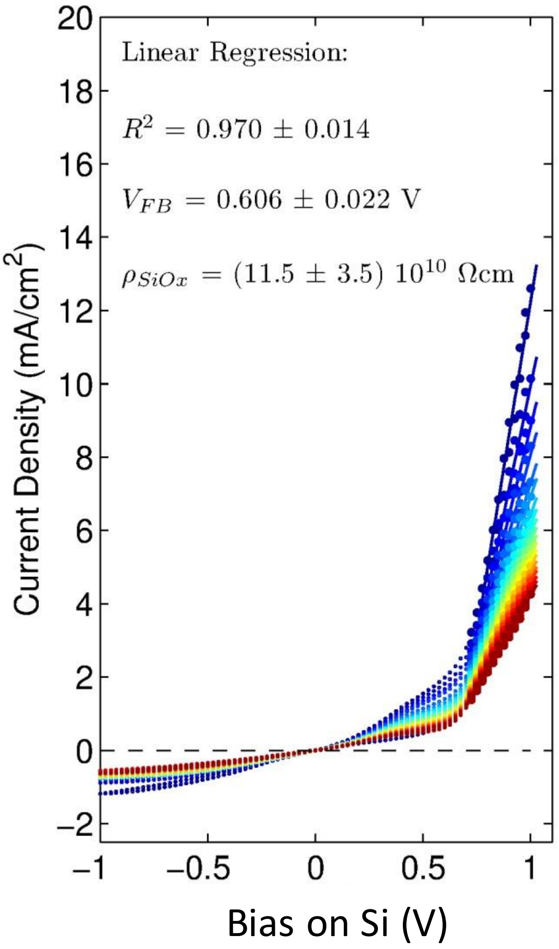

Figure S18. Current-voltage characteristics of bare *p*-type Si substrates with native SiO_x top contacted with EGaIn (**Si/SiOx**//GaOx/EGaIn.) At forward bias and $V_{fb} < V_{bias}$, a linear regression was used to determine V_{fb} and the resistivity of the native SiO_x was extracted (a thickness $d_{SiOx} = 0.6$ nm was assumed).

6.2- *J-V* characteristics and charge transport mechanisms

In a MmS interface, the most common mechanism used to describe the *J* - *V* response when the applied bias falls over the space charge region (semiconductor transport control) is the thermionic emission, assuming that the electrons and holes tunnel directly through the monolayer as

is generally the case for a monolayer thickness < 3.5 nm. The total effective barrier ϕ_{eff} considering the monolayer is given by:

$$\emptyset_{eff} = \emptyset_b + kT\beta l$$ **Equation S10**

$\emptyset_b$ is the Schottky barrier. *kTβl* describes the contribution of the interfacial layer to the effective barrier height, where *β* is the structure-dependent attenuation factor that depends on the tunneling mechanism, the nature of the monolayer and charge carriers type (holes for p-type Si or electrons for n-type Si); *l* is the thickness of the monolayer.[13,17–22]

Considering the effective barrier height ϕ_{eff}, the thermionic emission process occurs when the energy of some electrons or holes is enough to overload this barrier. Therefore, for the MmS interface, the current is proportional to exp($-\phi_{eff}/kT$). When the total series resistance of the device can be neglected, the expression for the current density is given by the **Equation S11**.[13,23,24]

$$J = A^*T^2 e^{\frac{-\phi_{eff}}{KT}} e^{\frac{qV}{nKT}} \left(1 - e^{\frac{-qV}{KT}}\right)$$ **Equation S 11**

where *V* is the applied bias voltage, *J* is the current density, *A** is the Richardson constant (110 and 32 $A \cdot K^{-2} \cdot cm^{-2}$ for *n*-type and *p*-type silicon, respectively), and *n* is the diode ideality factor which accounts for the non-idealities in the diode behavior. In the ideal case, *n* = 1.

Rearranging the **Equation S11** gives:

$$ln\left(\frac{J}{\left(1-e^{\frac{-qV}{KT}}\right)}\right) = \ln(A^*T^2) - \frac{\phi_{eff}}{KT} + \frac{qV}{nKT}$$ **Equation S12**

A plot of $ln\left(\frac{J}{\left(1-e^{\frac{-qV}{KT}}\right)}\right)$vs V should be ideally linear. n can be derived from the slope and ϕ_{eff} can be obtained from the intercept.

However, if the transport mechanism is not exclusively governed by the thermionic emission $n > 1$. These deviations can thus be attributed to the contribution of other factors, such as inhomogeneities in the barrier or series resistance.

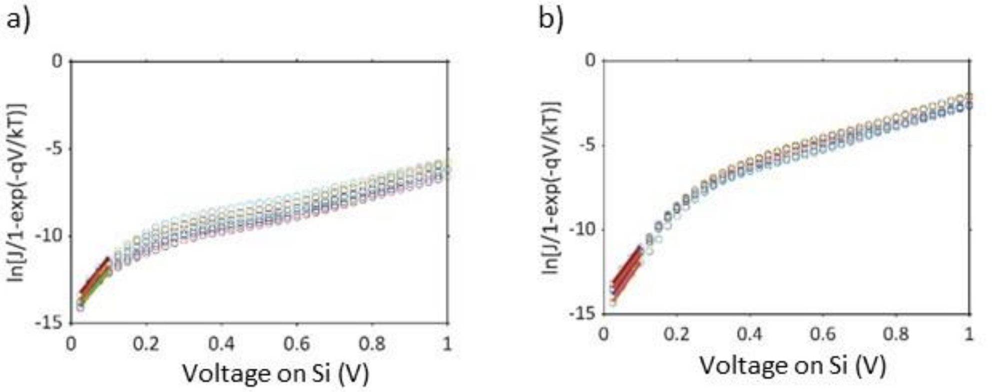

Figure S19. Determination of the effective barrier height using the **Equation S12**. a) Fit for 8 representative curves of the junction **Si/αH-PTM**//GaOx/EgaIn; ϕ_{eff} (**Si/αH-PTM**) = 0.74 ± 0.01 eV and n = 1.4 ± 0.1. b) Fit for 8 representative curves of the junction **Si/rad-PTM**//GaOx/EgaIn; ϕ_{eff} (**Si/rad-PTM**) = 0.74 ± 0.01 eV and n = 1.3 ± 0.1.

7-Photoresponse characterization

The photoresponse behaviour of the junctions was fitted using the following equation:

$$I_{pc} = \mathrm{S_{ph}}P + b \qquad \textbf{Equation S13}$$

where I_{pc} is the photocurrent, S_{ph} is the response factor (photosensitivity), P is the power density and b is the intercept.

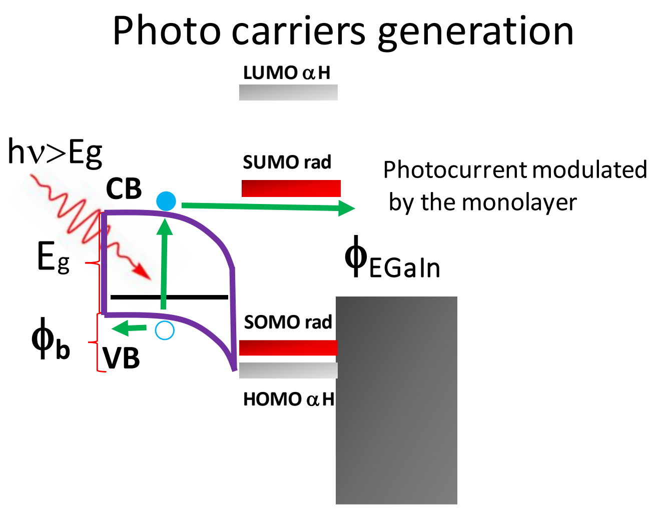

Figure S20. Schematic energy diagram for **Si/PTM**//GaOx/EgaIn under irradiation.

When a photon with an energy higher than the band gap interacts with a semiconductor, an electron/hole pair (e^-/h^+) is generated. For a p-type semiconductor, this process increases the density of electrons in the conduction band, whereas the density of holes is almost unaltered. However, only under depletion, an effective photo-effect can be observed because the density of minority carriers is significantly altered (here electrons). For p-type silicon, the driving force (electric-field) sweeps the electrons to the metal and the holes to the back-contact, leading to a photodiode-type functioning (**Figure S20**).

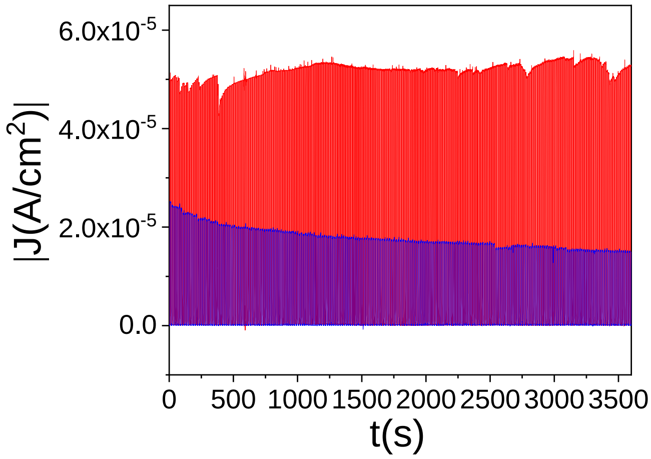

Figure S21. Stability of the junctions under 170 μW/cm^2 at 0 V bias voltage for 3600 seconds of ON (irradiation)/OFF (dark) cycles: **Si/αH-PTM** (blue) and **Si/rad-PTM** (red).

References

(1) Bejarano, F.; Olavarria-Contreras, I. J.; Droghetti, A.; Rungger, I.; Rudnev, A.; Gutiérrez, D.; Mas-Torrent, M.; Veciana, J.; Van Der Zant, H. S. J.; Rovira, C.; et al. Robust Organic Radical Molecular Junctions Using Acetylene Terminated Groups for C-Au Bond Formation. *J. Am. Chem. Soc.* **2018**, *140*, 1691–1696.

(2) Eckermann, A. L.; Feld, D. J.; Shaw, J. A.; Meade, T. J. Electrochemistry of Redox-Active Self-Assembled Monolayers. *Coord. Chem. Rev.* **2010**, *254*, 1769–1802.

(3) A.J. Bard; Faulkner, L. R. *Electrochemical Methods; Fundamentals and Applications*; John Wiley & Sons, Ltd, 2002.

(4) Chen, X.; Hu, H.; Trasobares, J.; Nijhuis, C. A. Rectification Ratio and Tunneling Decay Coefficient Depend on the Contact Geometry Revealed by in Situ Imaging of the Formation of EGaIn Junctions. *ACS Appl. Mater. Interfaces* **2019**, *11*, 21018–21029.

(5) Chiechi, R. C.; Weiss, E. A.; Dickey, M. D.; Whitesides, G. M. Eutectic Gallium-Indium (EGaIn): A Moldable Liquid Metal for Electrical Characterization of Self-Assembled Monolayers. *Angew. Chemie - Int. Ed.* **2008**, *47*, 142–144.

(6) Blum, V.; Gehrke, R.; Hanke, F.; Havu, P.; Havu, V.; Ren, X.; Reuter, K.; Scheffler, M. Ab Initio Molecular Simulations with Numeric Atom-Centered Orbitals. *Comput. Phys. Commun.* **2009**, *180*, 2175–2196.

(7) Adamo, C.; Barone, V. Toward Reliable Density Functional Methods without Adjustable Parameters: The PBE0 Model. *J. Chem. Phys.* **1999**, *110*, 6158–6170.

(8) Alcón, I.; Bromley, S. T. Structural Control over Spin Localization in Triarylmethyls. *RSC Adv.* **2015**, *5*, 98593–98599.

(9) Tkatchenko, A.; Scheffler, M. Accurate Molecular van Der Waals Interactions from

Ground-State Electron Density and Free-Atom Reference Data. *Phys. Rev. Lett.* **2009**, *102*, 073005.

(10) Kondo, M.; Mates, T. E.; Fischer, D. A.; Wudl, F.; Kramer, E. J. Bonding Structure of Phenylacetylene on Hydrogen-Terminated Si(111) and Si(100): Surface Photoelectron Spectroscopy Analysis and Ab Initio Calculations. *Langmuir* **2010**, *26*, 17000–17012.

(11) Sohn, K. E.; Dimitriou, M. D.; Genzer, J.; Fischer, D. A.; Hawker, C. J.; Kramer, E. J. Determination of the Electron Escape Depth for NEXAFS Spectroscopy. *Langmuir* **2009**, *25*, 6341–6348.

(12) Beamson, G.; Clark, D. T.; Kendrick, J.; Briggs, D. Observation of Vibrational Asymmetry in the High Resolution Monochromatized XPS of Hydrocarbon Polymers. *J. Electron Spectros. Relat. Phenomena* **1991**, *57*, 79–90.

(13) Faber, E. J.; De Smet, L. C. P. M.; Olthuis, W.; Zuilhof, H.; Sudhölter, E. J. R.; Bergveld, P.; Van Den Berg, A. Si-C Linked Organic Monolayers on Crystalline Silicon Surfaces as Alternative Gate Insulators. *ChemPhysChem* **2005**, *6*, 2153–2166.

(14) Wimbush, K. S.; Fratila, R. M.; Wang, D.; Qi, D.; Liang, C.; Yuan, L.; Yakovlev, N.; Loh, K. P.; Reinhoudt, D. N.; Velders, A. H.; et al. Bias Induced Transition from an Ohmic to a Non-Ohmic Interface in Supramolecular Tunneling Junctions with Ga2O3/EGaIn Top Electrodes. *Nanoscale* **2014**, *6*, 11246–11258.

(15) Silicon Dioxide Properties https://www.iue.tuwien.ac.at/phd/filipovic/node26.html.

(16) Pfattner, R.; Foudeh, A. M.; Chen, S.; Niu, W.; Matthews, J. R.; He, M.; Bao, Z. Dual-Gate Organic Field-Effect Transistor for PH Sensors with Tunable Sensitivity. *Adv. Electron. Mater.* **2019**, *5*, 1800381.

(17) Liu, Y. J.; Yu, H. Z. Alkyl Monolayer-Passivated Metal-Semiconductor Diodes: Molecular Tunability and Electron Transport. *ChemPhysChem* **2002**, *3*, 799–802.

(18) Liu, Y. J.; Yu, H. Z. Alkyl Monolayer Passivated Metal-Semiconductor Diodes: 2:** Comparison with Native Silicon Oxide. *ChemPhysChem* **2003**, *4*, 335–342.

(19) Selzer, Y.; Salomon, A.; Cahen, D. Effect of Molecule-Metal Electronic Coupling on through-Bond Hole Tunneling across Metal-Organic Monolayer-Semiconductor Junctions. *J. Am. Chem. Soc.* **2002**, *124*, 2886–2887.

(20) Selzer, Y.; Salomon, A.; Cahen, D. The Importance of Chemical Bonding to the Contact for Tunneling through Alkyl Chains. *J. Phys. Chem. B* **2002**, *106*, 10432–10439.

(21) Temircl, C.; Bati, B.; Sağlam, M.; Türüt, A. High-Barrier Height Sn/p-Si Schottky Diodes with Interfacial Layer by Anodization Process. *Appl. Surf. Sci.* **2001**, *172*, 1–7.

(22) Liu, Y. J.; Yu, H. Z. Molecular Passivation of Mercury-Silicon (p-Type) Diode Junctions: Alkylsilation, Oxidation, and Alkylsilation. *J. Phys. Chem. B* **2003**, *107*, 7803–7811.

(23) Sze, S. M.; Ng, K. K. *Physics of Semiconductor Devices*, 3rd ed.; John Wiley & Sons, Inc., 2006.

(24) Rhoderick, E. H.; Willians, R. H. *Metal-Semiconductor Contacts*, 2nd ed.; Oxford University Press, 1988.
